# Metagenomic profiling and temporal dynamics of antimicrobial resistance genes across hospital wastewaters in Dhaka, Bangladesh

**DOI:** 10.1128/spectrum.00755-26

**Published:** 2026-10-01

**Authors:** Md. Rubiath Islam, Rajib Sarkar, Subarna Sandhani Dey, Isthiaque Ahmed, Mohammad Robel Hossen Patwary, Zafrul Hasan, Moshiul Alam Mishu, Monzur Morshed Ahmed, Abhijit Chowdhury

**Affiliations:** 1 Food Microbiology Research Laboratory, Institute of Food Science and Technology, Bangladesh Council of Scientific and Industrial Research 130051, Dhaka, Bangladesh; 2 Department of Biochemistry and Molecular Biology, Shahjalal University of Science and Technology 113074, Sylhet, Bangladesh; 3 Central Analytical and Research Facilities, Bangladesh Council of Scientific and Industrial Research 130051, Dhaka, Bangladesh; Connecticut Agricultural Experiment Station, New Haven, Connecticut, USA

**Keywords:** hospital wastewater, antimicrobial resistance, metagenomics, integron, temporal dynamics

## Abstract

Hospital wastewater (HWW) in Bangladesh is often discharged into municipal sewage and water bodies without adequate treatment. It raises concern that hospitals may act as reservoirs and amplification points for clinically important antimicrobial resistance genes (ARGs). However, culture-independent data on the composition, mobility, and seasonal behavior of these genes in Bangladeshi hospital wastewater remain limited. Here, we combined single monsoon time-point baseline shotgun metagenomic sequencing with longitudinal qPCR to characterize wastewater from three tertiary hospitals in Dhaka and a nearby community wastewater line across three seasonal sampling points. Across sites based on baseline metagenomic profiling, the putative resistome was dominated by aminoglycoside, sulfonamide, macrolide–lincosamide–streptogramin (MLS), tetracycline, and β-lactam resistance, primarily associated with Actinomycetota and Pseudomonadota. Hospital effluents contain putative clinically important genes, including the *vanA* cluster, *blaVIM*, *blaOXA-232*, *blaIMP*, and *blaKPC*. Metagenomic analysis linked many putative ARGs to plasmid-predicted contigs, transposition, or integration modules, while virulence profiling showed enrichment of putative genes related to adhesion, motility, iron acquisition, and secretion, particularly in MHWW-2. Exploratory network co-occurrence from baseline metagenomics primarily suggested fecal-derived gut commensals, alongside pathogens, as reservoirs and putative vectors for ARG input into the wastewater system. Longitudinal qPCR showed higher absolute loads of selected ARGs at the pre-winter sampling point, and the class 1 integron gene (*intI1LC*) tracked total ARG burden across seasonal transitions. These findings identify hospital wastewater in Dhaka primarily as a dynamic reservoir of clinically important and potentially mobile resistance determinants, and support its use as a target for wastewater-based AMR surveillance.

**IMPORTANCE:**

Wastewater surveillance can provide early warning of AMR, but in Bangladesh, it is rarely applied beyond culture or single-gene PCR, limiting detection of emerging, mobile, and facility-specific threats. This study integrates metagenomics with longitudinal qPCR to connect which resistance genes are present with who carries them and how they change seasonally across multiple hospitals. We show that hospital effluents contain a shared background resistome yet disproportionately concentrate last-line resistance in taxa that include clinically important lineages, with signals rising toward pre-winter. By placing high-risk putative ARGs in putative plasmid, integron, and transposase contexts and alongside virulence repertoires from baseline metagenomic profiling, our results identify plausible genomic mechanisms that may contribute to spread beyond hospitals. These findings position hospital effluents in Dhaka preliminarily as dynamic reservoirs of clinically important, mobile AMR and support wastewater-based epidemiology for sustained surveillance and risk mitigation.

## INTRODUCTION

Antimicrobial resistance (AMR) is among the 10 most significant threats to human health and results in hundreds of thousands of fatalities annually (1). Bangladesh is a low- and middle-income country (LMIC) in South Asia, where antibiotic-resistant infections are prevalent among both hospitalized patients and the general population (2, 3). From 2016 to 2023, Bangladesh had an 11% increase in AMR alongside an 82% reduction in the efficacy of routinely used antibiotics (4). A combination of unregulated over-the-counter sales, self-medication, systemic healthcare deficiencies, and irrational prescribing practices among practitioners propels the extensive abuse of antibiotics (5, 6). The capital of Bangladesh, Dhaka, has a population of over 16 million, placing heavy pressure on government hospitals and contributing to a rising burden of nosocomial infections (3, 7).

Hospital sewage systems function as critical hotspots for AMR, receiving substantial loads of antibiotic residues, biocides, and patient excrement that drive the selection and spread of resistant pathogens (8, 9). In Bangladesh, several studies investigated the presence of ARGs in HWW. Several of these studies have concentrated on specific pathogenic bacteria such as *Escherichia coli*, *Staphylococcus aureus*, *Klebsiella pneumoniae*, *Pseudomonas aeruginosa*, *Bacillus cereus*, and *Aeromonas* spp. (10–16). Some studies involve genotypic evaluation or a combination of genotypic and phenotypic assessment (12, 17–19). Many of these studies demonstrated that HWW serves as a crucial and distinctive reservoir of antibiotic resistance, exhibiting a substantially higher prevalence of ubiquitous genes, including *sul1*, *tetA*, *mecA,* and ESBLs (10–13, 20). Recently, *mcr* variants have also been identified in hospital effluents across Bangladesh and India (21, 22). Numerous studies have demonstrated a strong genetic linkage between clinically important ARGs and the class 1 integrase gene (*intI1*), highlighting the role of these mobile genetic elements in spreading multidrug resistance (23–26). Moreover, HWW serves as a hyper-concentrated locus that intensifies selection pressure, facilitating the horizontal transmission of resistance genes (27). The consequences of these measures and inadequate data on resistance trends in Bangladesh highlight an urgent need for improved surveillance of resistance in hospital settings.

In developing nations, including Bangladesh, HWW is discharged into the municipal sewage system, and much of this wastewater is not effectively intercepted before reaching canals or rivers, creating conditions that may facilitate the spread of multidrug-resistant (MDR) organisms in the surrounding environment (28–31). A recent study showed that many hospitals in Dhaka lack effective treatment infrastructure, with some systems in disrepair, resulting in hazardous liquid waste being discharged directly into the environment (32). Waterlogging during the monsoon causes these contaminated waters to come into closer contact with surrounding communities. In Dhaka, the Water Supply and Sewerage Authority (WASA) treats river water for municipal use, yet studies have reported fecal coliform and *E. coli* contamination in WASA-supplied water and the presence of *blaNDM-1* in tap water (33–35). These findings suggest a plausible route through which inadequately treated effluent contaminates connected surface waters and increases downstream exposure risk through flooding, unsafe household water use, and other wastewater-impacted environments.

Despite increasing concern about AMR in HWW, current surveillance in Bangladesh remains limited to culture-dependent methods and targeted PCR assays, leaving much of the broader resistome and virulome unresolved. Wastewater-based AMR surveillance studies emphasize that stronger inference requires time and location-matched sampling and longer follow-up rather than short campaigns (36). To date, there is a distinct paucity of comprehensive, culture-independent studies to profile the total resistome and virulome of hospital effluents in this region. Furthermore, there is a lack of data regarding the persistence and stability of high-risk resistance genes over extended periods, which is crucial for understanding the long-term ecological impact of untreated HWW. To monitor the AMR threat, wastewater-based epidemiology (WBE) has emerged as a powerful, non-invasive surveillance method (37). Traditional surveillance relies on clinical samples that require informed consent and frequently overlook asymptomatic carriers, whereas WBE offers an extensive overview of resistance levels within the population (38, 39). In this study, we combined shotgun metagenomic sequencing and targeted quantitative real-time PCR to investigate the composition and seasonal dynamics of AMR in hospital wastewater from Dhaka. Our analysis focused on microbial diversity, ARGs, virulence factors, and selected mobile genetic elements, together with their distribution across sampling sites and time points. This study provides a culture-independent baseline view of the hospital wastewater resistome in Bangladesh and targeted longitudinal surveillance of selected high-risk markers across seasons.

## RESULTS

### Sample collection

We collected HWW from June to November 2025 from three government-run tertiary care hospitals in southern Dhaka, namely Bangladesh Medical University Hospital (HWW 1), Dhaka Medical College Hospital (HWW 2), and Mitford Medical Hospital (HWW 3), along with a community sewage sample from Azimpur. Dhaka Medical College Hospital and Mitford Medical Hospital are located in densely populated Old Dhaka and serve the highest patient volumes (Table 1). Because these facilities provide low-cost care, they receive a large influx of low-income patients (7). At each hospital, we collected wastewater from three locations along the sewer pipeline to capture spatial heterogeneity across the hospital catchment. These sub-samples were subsequently pooled and homogenized to produce a single composite sample for downstream analyses.

**TABLE 1:** Information of the sampling sites^*a*^

Sample collection details	HWW-1	HWW-2	HWW-3	ACWW
Collection site	Bangladesh Medical University Hospital	Dhaka Medical College Hospital	Mitford Medical Hospital	Azimpur Community
Coordinate	23.7391°N, 90.3950°E	23.7248°N, 90.3971°E	23.7110°N, 90.4010°E	23.7252°N, 90.3859°E
Number of beds/populations	1,900	2,600	900	>96,000
Sampling time	June, September, November	June, September, November	June, September, November	June, September, November

^*a*^ ACWW refers to Azimpur Community Wastewater. The prefix “M” was used to denote the monsoon HWW samples collected in June and used for baseline shotgun metagenomic analysis.

### Taxonomic structure (single monsoon time-point baseline shotgun metagenomic sequencing)

**Fig 1:**
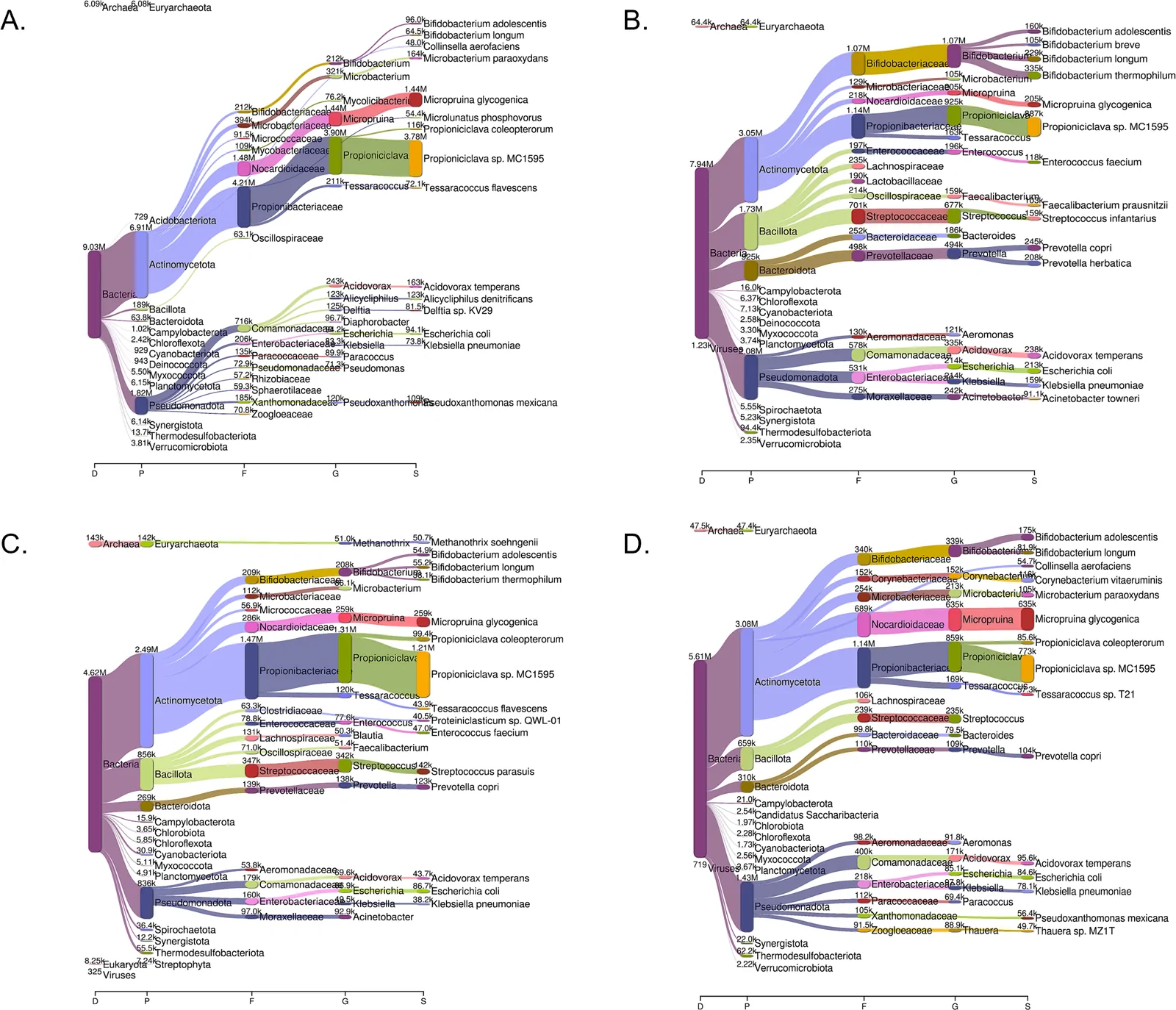
Taxonomic classification of the wastewater microbiome in terms of read distribution: (**A**) MHWW-1, (**B**) MHWW-2, (**C**) MHWW-3, and (**D**) ACWW.

**Fig 2:**
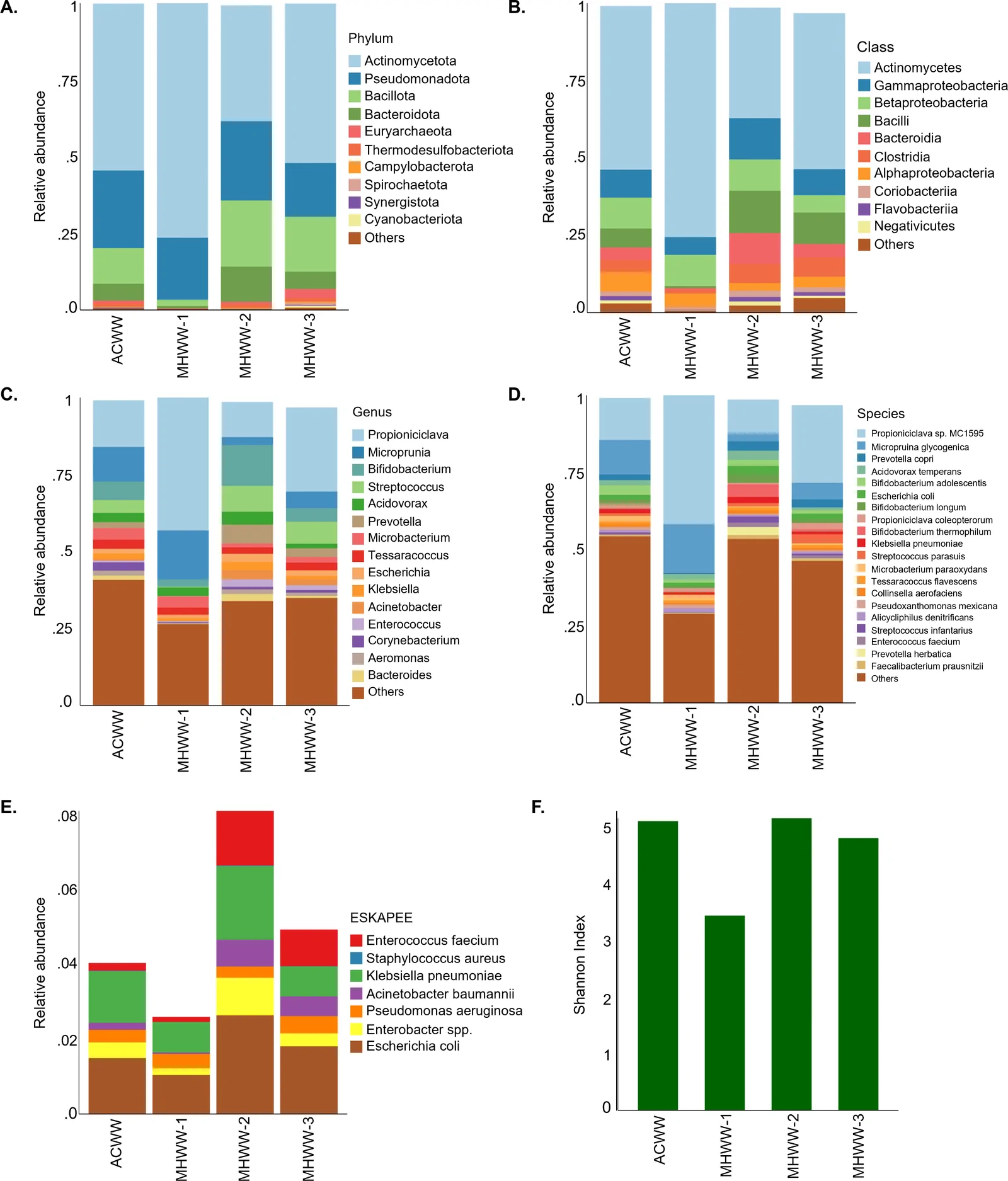
Relative abundance of the bacteria and their diversity at different taxonomic levels: (**A**) phyla, (**B**) class, (**C**) genus, (**D**) species, and (**E**) abundance of ESKAPEE pathogen. (**F**) Alpha diversity of the microbiome calculated by Shannon index method.

Across samples, we detected 37 phyla, 80 classes, 1,311 genera, and 4,849 species (Fig. 1). The microbiome was consistently dominated by Actinomycetota (37.8–76.4%), with Pseudomonadota as the second most abundant phylum (17.5–25.8%). Bacillota and Bacteroidota were most enriched in MHWW-2 with 21.5% and 11.5%, respectively (Fig. 2A). At the class level, Actinomycetes prevailed (35.7–75.5%), whereas MHWW-2 showed higher Bacilli (13.7%), Gammaproteobacteria (13.3%), and Bacteroidia (10.0%) (Fig. 2B). At the genus level, *Propioniciclava* and *Micropruina* formed a core signal, with MHWW-1 highly skewed toward *Propioniciclava* (43.1%). MHWW-2 displayed a broader, clinically relevant profile. At the species level, *Propioniciclava* sp. MC1595 dominated all samples (11.0–41.9%), followed by *Micropruina glycogenica* (Fig. 2C and D). HWW is often enriched with highly resistant, potentially pathogenic bacteria, including ESKAPEE organisms (40). These pathogens were detectable in all of our samples but were most elevated in MHWW-2, which carried *Escherichia coli* (2.64%), *Klebsiella pneumoniae* (1.97%), *Enterococcus faecium* (1.46%), and *Acinetobacter baumannii* (0.72%) (Fig. 2E). According to the Shannon diversity index, MHWW-1 has the lowest diversity, indicating a community strongly dominated by a few species, while the rest of the samples were much more even and diverse (Fig. 2F).

### Prevalence of putative ARGs in wastewater (single monsoon time-point baseline shotgun metagenomic sequencing)

**Fig 3:**
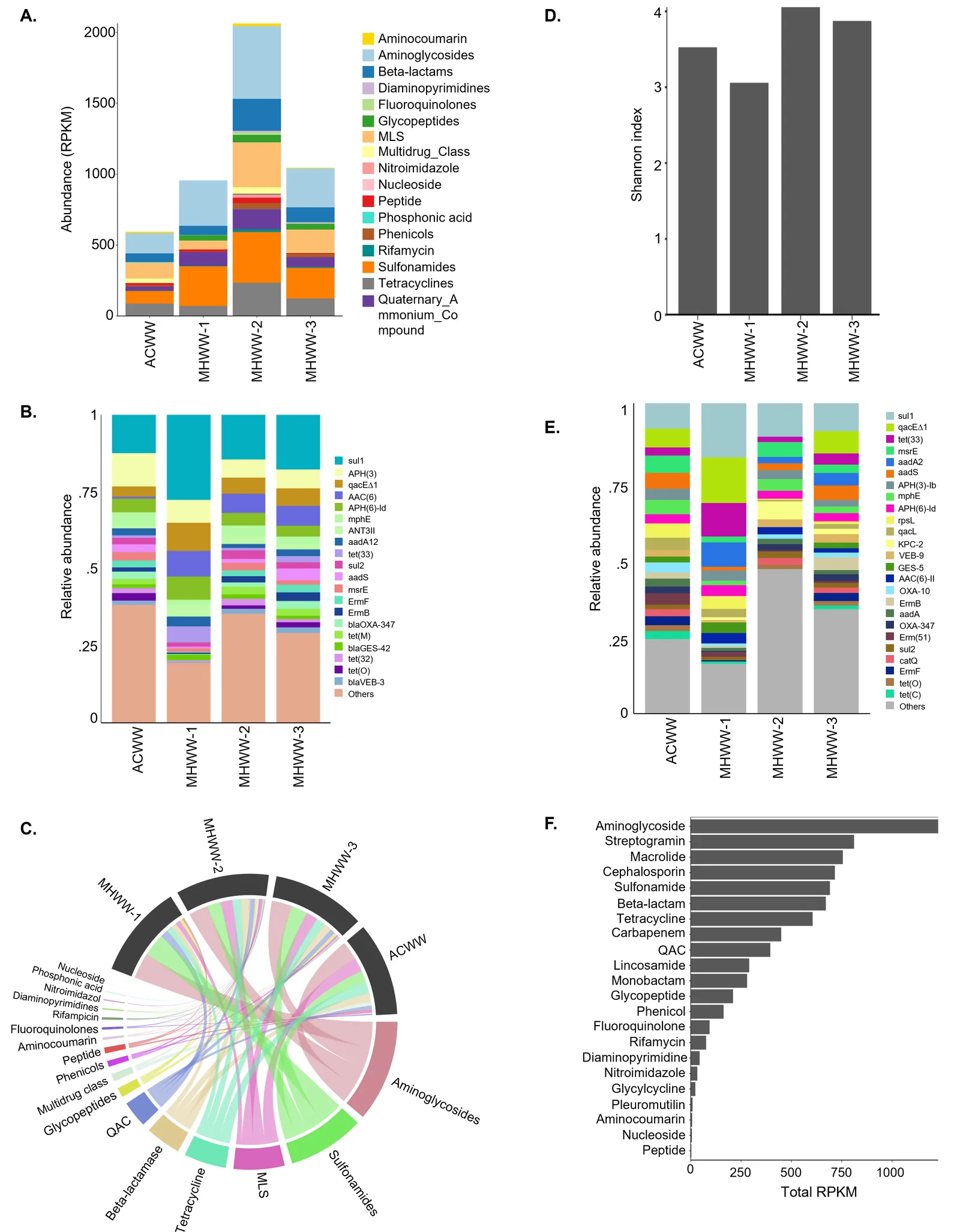
Class-level and gene-level resistome profiles in community and HWW. (**A**) Absolute ARG abundance (RPKM) by antibiotic class identified by ShortBRED. (**B**) Relative abundance of the most abundant ARG subtypes in ShortBRED. (**C**) Distribution of antibiotic resistance classes across sites in relative abundance. (**D**) Shannon diversity index of ARG profiles. (**E**) Relative abundance of clinically important ARG subtypes identified by RGI. (**F**) Ranking of resistance classes by total RPKM across all samples.

We identified a total of 218 non-redundant putative AMR genes by ShortBRED and quantified as RPKM (Table S2). Compared with ACWW, all three HWW sites showed higher overall putative ARG burdens, with MHWW-2 exhibiting the highest total abundance (Fig. 3A). Despite site-to-site differences in abundance, the putative resistome composition was broadly conserved at the class level (Fig. 3C). Across all samples, putative aminoglycosides, sulfonamides, MLS, tetracyclines, and β-lactams contributed about 82% of total ARG abundance. At the gene level, the high prevalence and abundance of putative *sul1* together with *qacEΔ1* were consistently found in the wastewater network (Fig. 3B). High burdens of putative aminoglycoside-modifying enzymes including *AAC*(6) (0.62–8.30%), *APH(3*) (5.90–10.79%), and *APH(6)-Id* (3.51–7.56%) indicate substantial potential to compromise clinically important aminoglycosides. At the same time, tetracycline resistance was driven by putative *tet(33*). HWW often contain more clinically important ARGs than the community wastewater (41). Here, we found that the HWW resistome contains putative glycopeptide resistance genes (approximately 1.88–3.64%) and carbapenemase genes, including *blaVIM*, *blaOXA-232*, *blaIMP*, and *blaKPC*. The mobile colistin resistance putative *mcr-5* was also detected in MHWW-2 and MHWW-3. The putative tetracycline-inactivating enzyme gene *tet(X*) was detected at low abundance across all four sites.

In contig-based RGI analysis, we identified 132 unique putative ARGs spanning 23 antibiotic classes and quantified them as RPKM (Table S3). At the class level, ShortBRED and RGI converged on the same dominant resistance landscape (Fig. 3B and E), with aminoglycoside resistance contributing the highest total ARG abundance, followed by MLS and β-lactam resistance (Fig. 3F). Importantly, RGI resolved multiple high-concern β-lactamase variants that were less explicit in marker-based profiles. MHWW-2 showed the broadest putative resistome and the highest overall β-lactam burden (14.13%) to which *blaOXA-205* (1.97%), *blaKPC-2* (1.50%), *blaNPS-1* (1.41%), *blaNDM-1* (1.26%), *blaOXA-1011* (1.12%), and *blaCTX-M-15* (0.79%) were among the major contributors. Multiple additional markers, including putative *blaGES-5*, *blaOXA-181*, and *blaIMP-26* and *IMP-62*, were detected only at this site (Table S3). The prototype variant of the New Delhi metallo-β-lactamase putative *blaNDM-1* is frequently found on mobile genetic elements, facilitating their dissemination (42, 43). We detected putative *blaNDM-1* on plasmid-predicted contigs and in proximity to transposition/integration modules, supporting a mobile genetic context in this site, although direct experiments of horizontal transfer were not performed in this study. MHWW-1 was dominated by putative *blaOXA*-type enzymes and *blaVIM-12* (0.58%), with *blaOXA-1016* (1.25%) and *blaNPS-1* (0.84%) as the largest contributors. MHWW-3 retained clinically important putative β-lactam markers, including *blaKPC-2* (0.67%), *blaVIM-4* (0.28%), and *blaCTX-M-15* (0.52%).

**Fig 4:**
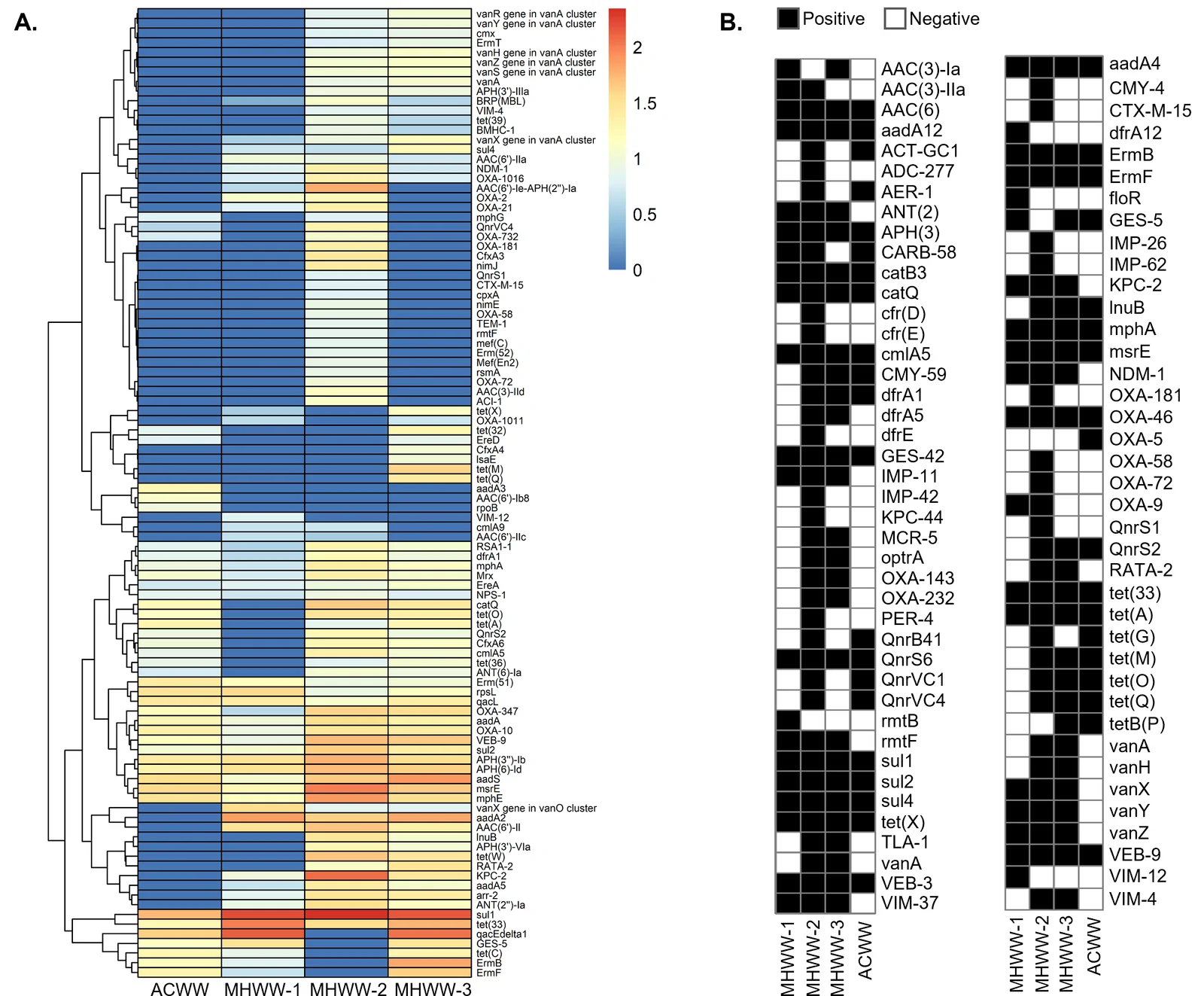
Resistome composition across community and hospital wastewater. (**A**) Clinically important ARGs detected from assembled contigs using CARD/RGI, showing normalized gene abundance (log-transformed RPKM) across samples. Genes were hierarchically clustered based on their abundance profiles. (**B**) Presence/absence matrix of key high-priority ARGs across the same samples.

RGI also captured mutation in *rpsL* and *rpoB* and identified a complete putative *vanA* gene cluster in MHWW-2 and MHWW-3, while no vanA-cluster genes were detected in ACWW (Fig. 4A). Shannon diversity was highest in MHWW-2 and MHWW-3, whereas MHWW-1 and ACWW exhibited lower heterogeneity, indicating reduced evenness and richness in those communities (Fig. 3D). Previous studies have identified several ARGs as clinically important, as they are associated with treatment failure in hospitals and with MGEs (44). We have identified putatively many of these genes in MHWW-2 and MHWW-3. In comparison, the ACWW has the lowest detectable level of these genes (Fig. 4B). Interestingly, many of these genes are also identified putatively as top abundant genes in both ShortBRED and RGI-based analyses, indicating their high abundance in Bangladeshi HWW. In contrast, ACWW carried a high-abundance putative core resistome, including *sul1* (8.21%), macrolide determinants *mrx* (5.56%) and *mphA* (4.60%), tetracycline resistance genes including tet(A) (2.81%), tet(C) (2.61%), and tet(O) (1.85%), and a few detectable β-lactamases, including *blaCTX-M-15* (0.89%) and *blaOXA-58* (0.65%).

### Genomic co-occurrence of putative ARGs and putative MGEs (single monsoon time-point baseline shotgun metagenomic sequencing)

We identified co-localization of putative ARGs on individual contigs, including multiple clinically important ARGs. Approximately one-third of all detected putative ARGs were assigned to plasmid-predicted contigs, with multi-ARG contigs suggesting that resistant determinants may preferentially cluster on potentially mobile genetic backbones. In combination with the detection of plasmid replicon markers and plasmid-associated maintenance or transfer genes, these results support the presence of putatively plasmid-associated ARGs. Across all samples, putative 49 multi-ARG contigs were identified. Among them, 16 were restricted to a single drug class, whereas the remaining contigs encoded resistance to multiple drug classes, indicating frequent co-localization of multidrug resistance within the same genetic unit. MHWW-2 accounted for nearly half of these putative multi-ARG contigs, seven of which were associated with resistance to the same drug class. Within the multi-class assemblies, the most recurrent co-occurrence patterns linked putative β-lactam resistance with aminoglycoside or macrolide resistance, highlighting common multidrug linkage structures. Putative β-lactamase genes in HWW were more often associated with plasmid-predicted contigs than those in ACWW. In MHWW-2, putative *blaCTX-M-15* and *blaCMY-4* were found to be plasmid associated, while putative *blaVEB-9* was consistently chromosome-associated across samples, together indicating primarily a persistent cephalosporin-resistance reservoir with implications for treatment failure (45).

**Fig 5:**
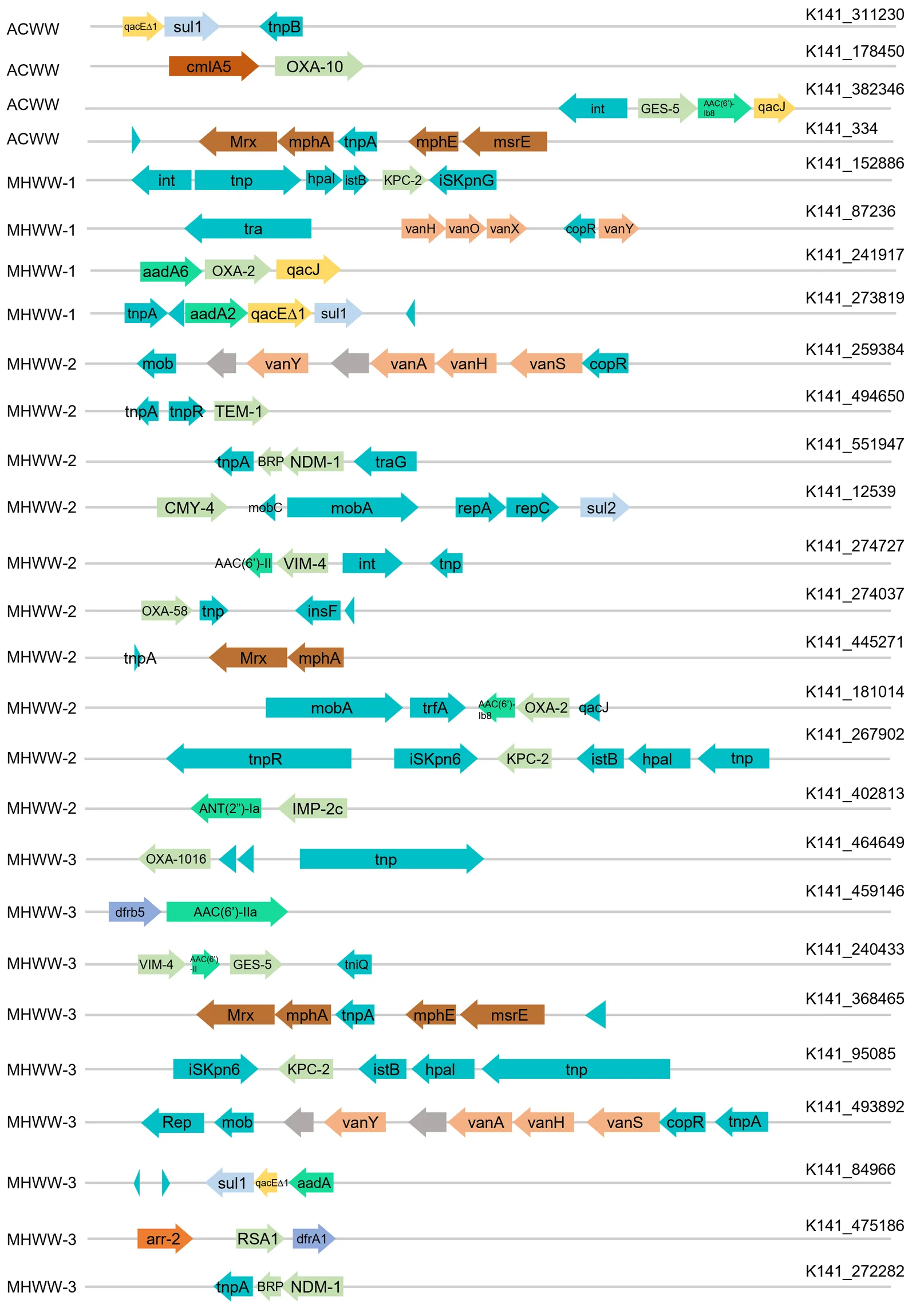
Genetic context of clinically important antibiotic resistance genes across community and hospital wastewater assemblies.

Interestingly, clinically important putative ARGs were frequently embedded within putative MGEs (Fig. 5). In ACWW, resistance features were dominated by putative class 1 integron-like signatures including *qacEΔ1–sul1* with *tnpB*, alongside a contig carrying *blaGES-5* co-localized with *int* and *qacJ*. This region is part of the conserved segment commonly associated with class 1 integrons and is widely used as an indicator of integron-linked resistance in anthropogenically impacted systems (46). In contrast, HWW contigs showed a broader repertoire of putative high-risk determinants and clear mobility potential, including putative carbapenemases such as *blaKPC-2*, *blaNDM-1*, *blaVIM-4*, and *blaIMP-2c* positioned adjacent to putative transposition/integration modules such as *tnp*, *tnpA*, *tnpR*, and *int*, and insertion sequences including *ISKpn6* and *iSKpnG*. This supports preliminarily potential mobilizable carbapenem resistance platforms. Several contigs also carry plasmid maintenance and transfer features, such as putative *mob*, *rep*, *trfA*, and *tra*, and β-lactamases such as *blaTEM-1* and *blaCMY-4* appeared within these plasmid-associated backbones. Both MHWW-2 and MHWW-3 contain a complete putative *vanA* operon module, consistent with a transferable glycopeptide resistance locus. Co-localization patterns also supported co-selection. Disinfectant/biocide-associated putative *qac* genes frequently co-occurred with putative ARGs, often alongside *sul1*, macrolide, and aminoglycoside markers, consistent with integron-like regions that can be maintained under non-antibiotic pressures common in wastewater environments.

**Fig 6:**
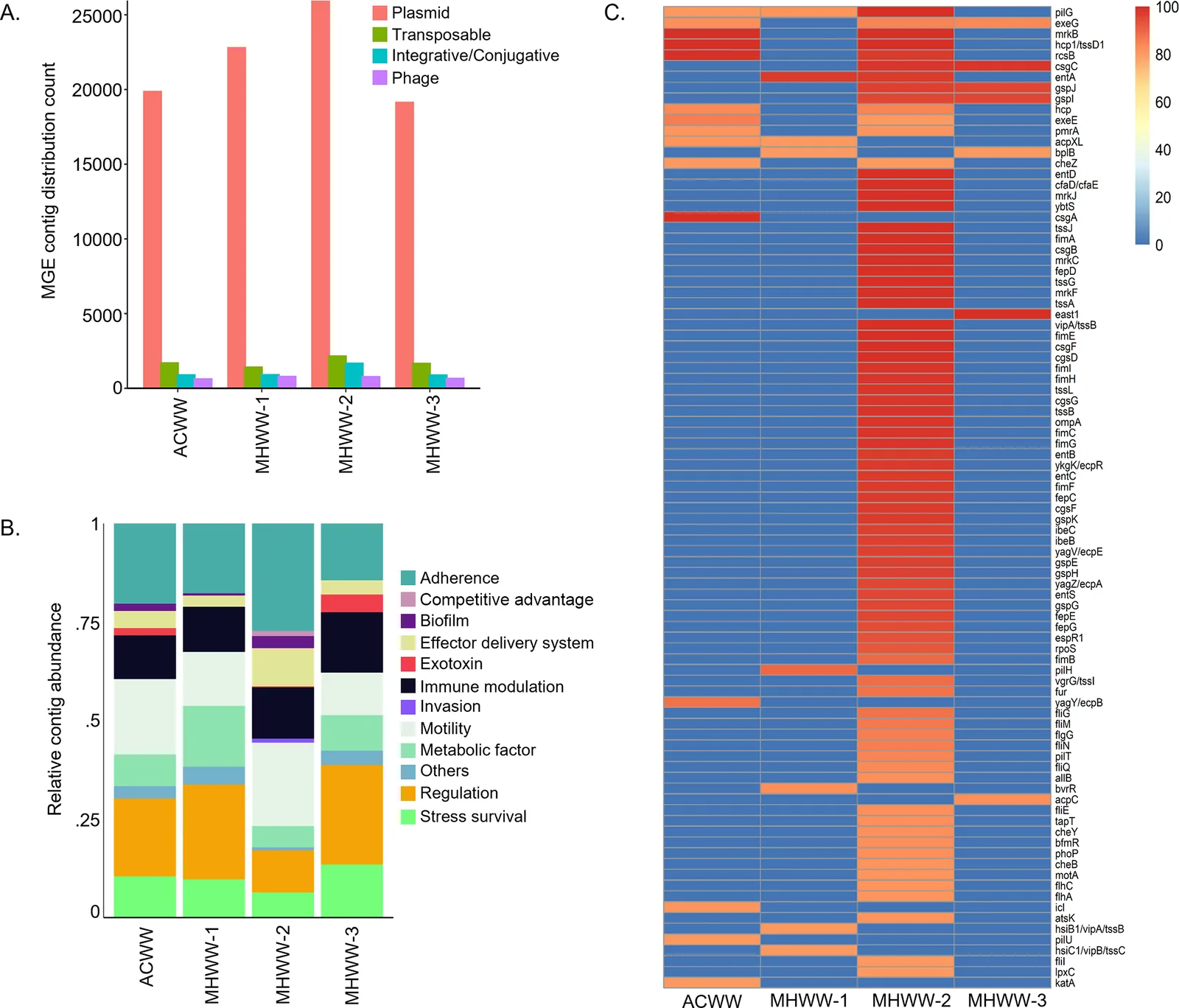
Mobile genetic elements and virulence-factor profiles across community and hospital wastewater samples. (**A**) Distribution of MGE-associated contigs. (**B**) Relative composition of virulence factors by functional class. (**C**) Heatmap of representative virulence genes across samples, with color indicating gene-level match.

In plasmid identification analysis, 29 putative plasmid replicon markers were identified across all the samples corresponding to 16 unique *Inc* groups and Col-type plasmids (Table S4). MHWW-2 showed the most complex putative plasmid profile with 18 distinct markers including multiple incompatibility groups. Putative *IncQ1* and *IncP(6*) were identified in all four samples, predicting a stable core mobilome. These plasmids are noted for efficient mobilization and broad host range, supporting their role as inter-host shuttles for resistance determinants in mixed wastewater communities (47). Putative plasmid-associated functions were abundant in every sample, while transposable elements, integrative and conjugative element (ICE), and phage-associated genes were consistently detected primarily (Fig. 6A).

### Distribution and abundance of putative VFs (single monsoon time-point baseline shotgun metagenomic sequencing)

VFs can act as ecological connectivity traits that promote persistence across niches by enhancing adhesion, motility, secretion, and biofilm formation, thereby indirectly supporting the maintenance and spread of AMR in complex wastewater environments (48, 49). In this study, we identified more than 200 unique putative VFs (Table S5), primarily associated with motility and surface attachment-related determinants, including type IV pilus, twitching motility components such as *pilG*, *pilI*, *pilT*, and *pilQ*, flagellar proteins, and chemotaxis proteins such as *fli* and *flg* genes, and *motA*, *cheY* (Fig. 6B). Surface-associated virulence proteins can mediate adhesion, invasion, immune evasion, and biofilm formation, which directly increases infection potential (50). MHWW-2 was enriched for putative adhesion and biofilm-associated modules, including type 1 fimbriae, curli-related factors, and additional fimbrial and biofilm-associated genes, including *mrkB/mrkC* and pilus-associated loci such as *ecp/cfa* components. It also carried strong signatures of putative iron acquisition, particularly the enterobactin system *entC/entD/entS* with uptake components such as *fepG*, consistent with enhanced fitness under nutrient limitation. Interestingly, secretion-related putative VFs were also concentrated in MHWW-2, including T2SS proteins and multiple T4SS components, indicating a higher potential for interbacterial competition and host-interaction capacity. In contrast, MHWW-3 showed fewer high-confidence loci but contained a putative *east1*-toxin-associated signal (Fig. 6C).

### Predicted taxonomic hosts of putative ARGs (single monsoon time-point baseline shotgun metagenomic sequencing)

Identification of the taxonomic reservoir of ARGs is essential for understanding how resistance emerges and persists across wastewater niches (22). Therefore, the RGI results were linked to the taxonomic lineage. Across all sites, putative ARGs were primarily associated with Actinomycetota and Pseudomonadota (Fig. S1A). ACWW and MHWW-1 were dominated by Actinomycetota-associated putative ARGs, with approximately 45% and 62%, respectively. MHWW-2 showed a substantial shift toward Pseudomonadota and Bacillota, with 34% and 31%, respectively. This site-specific shift is consistent with wastewater resistomes reflecting varying contributions from environmental wastewater-associated taxa versus human-associated microbiota and healthcare-associated inputs, which can restructure both the microbiome and ARG host landscape (51, 52). At the genus level, putative ARGs in ACWW and MHWW-1 were largely assigned to environmental wastewater-associated genera such as *Propioniciclava* and *Micropruina*, with contributions from *Microbacterium* and *Streptomyces* and low-to-moderate contributions from *Enterobacteriaceae*. MHWW-2 exhibited the highest putative ARG diversity concentrated in clinically relevant genera, including *Escherichia*, *Enterococcus*, *Streptococcus*, *Acinetobacter*, and *Bacteroidales*. Multiple last-line resistance putative markers were linked to Pseudomonadota-affiliated contigs. For example, putative *blaNDM-1* often resolved only to Gammaproteobacteria, whereas putative *blaCTX-M-15*, *blaOXA-181*, and *blaIMP-26* resolved to Enterobacterales. Interestingly, putative *blaIMP-62* found in MHWW-2 was assigned to *Pseudomonas*. Putative glycopeptide resistance gene *vanA-*cluster components were primarily associated with *Enterococcus* in MHWW-2 and MHWW-3 (Fig. S1B).

**Fig 7:**
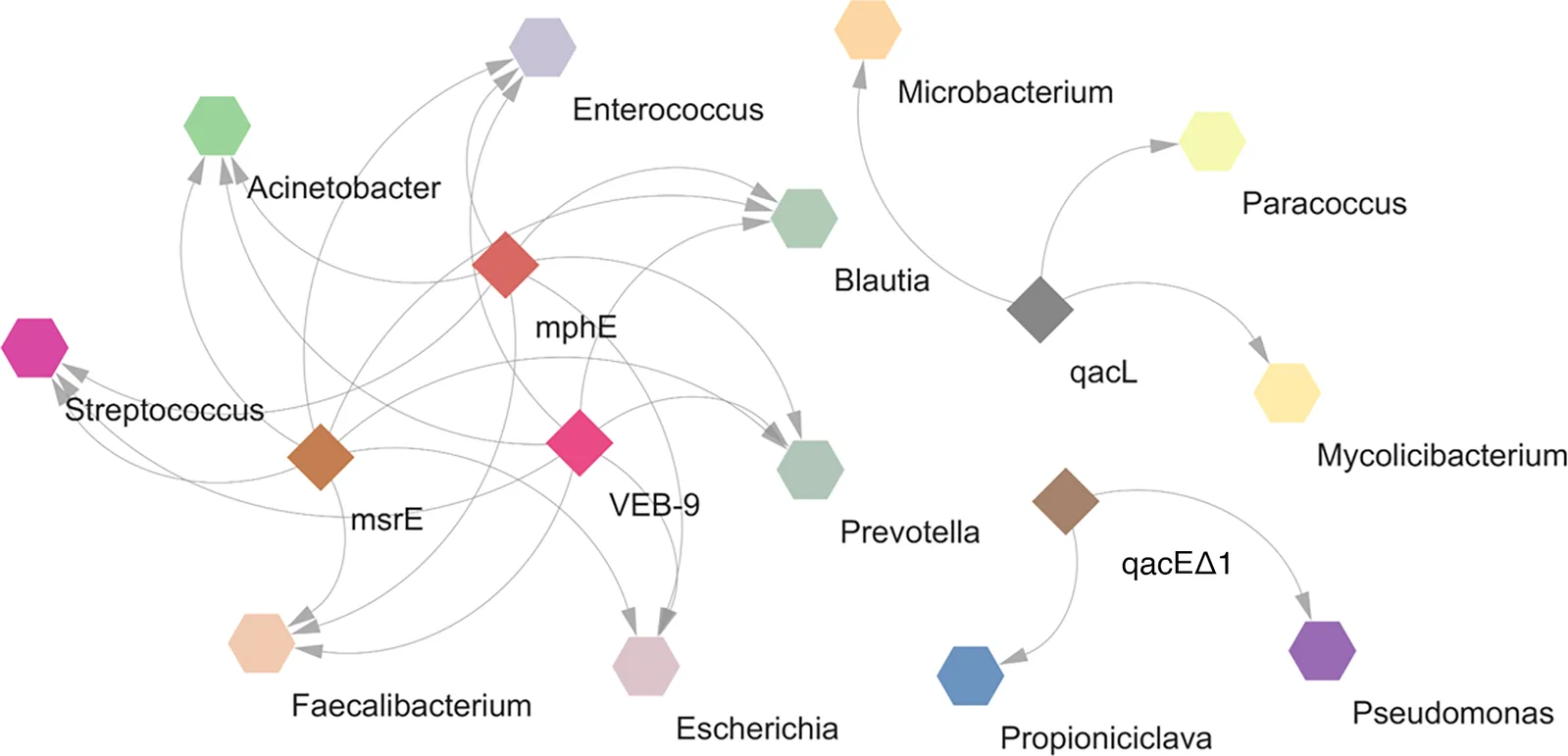
ARG-taxonomy co-occurrence network. The network analysis shows the co-occurrence of the top 20 ARG subtypes across top genera. The link pattern denotes Spearman’s correlation with a cutoff value of 0.8 (ρ value). Based on *n* = 4 baseline metagenomic samples (single monsoon timepoint per site). Presented as an exploratory, descriptive visualization given the limited sample size and not intended to support formal statistical inference of ARG-taxon association.

The non-random co-occurrence of ARGs with specific taxa can serve as a plausible proxy for putative host or reservoir signals (53). We explored the co-occurrence patterns between the abundance of the top 20 ARG subtypes across the baseline metagenomic samples (*n* = 4 sites, single monsoon timepoint) and the associated bacterial abundance at the genus level (Fig. 7). Given this limited sample size, network analysis was used as an exploratory, hypothesis-generating tool to examine co-occurrence patterns between ARG subtypes and bacterial taxa, rather than to support formal statistical inference of association strength. Previous studies have suggested that non-random co-occurrence patterns between ARGs and microbial taxa can provide possible host information when ARGs and coexisting taxa show similar abundance trends (22). In our analysis, the retained positive ARG–genus associations linked several clinically relevant putative ARGs, including the β-lactamases (*blaOXA-10*, *blaOXA-347*, *blaVEB-9*), the aminoglycoside resistance gene (*aadA*), the MLS resistance genes (*erm(51*), *mphE*, *msrE*), the biocide-associated genes (*qacEΔ1*, *qacL), catQ,* and *rpsL*, with both gut-associated and wastewater-associated genera. Genera identified in the exploratory network included *Escherichia*, *Enterococcus*, *Pseudomonas*, *Streptococcus*, *Prevotella*, *Blautia*, *Faecalibacterium*, *Acinetobacter,* and *Propioniciclava*. These associations were broadly consistent with the contig-level taxonomic assignments, including links to clinically relevant groups such as *Enterobacterales*, *Pseudomonas*, and *Enterococcus*, and were therefore putative hosts.

### Metagenomic assembled genome (MAG) (single monsoon time-point baseline shotgun metagenomic sequencing)

**Fig 8:**
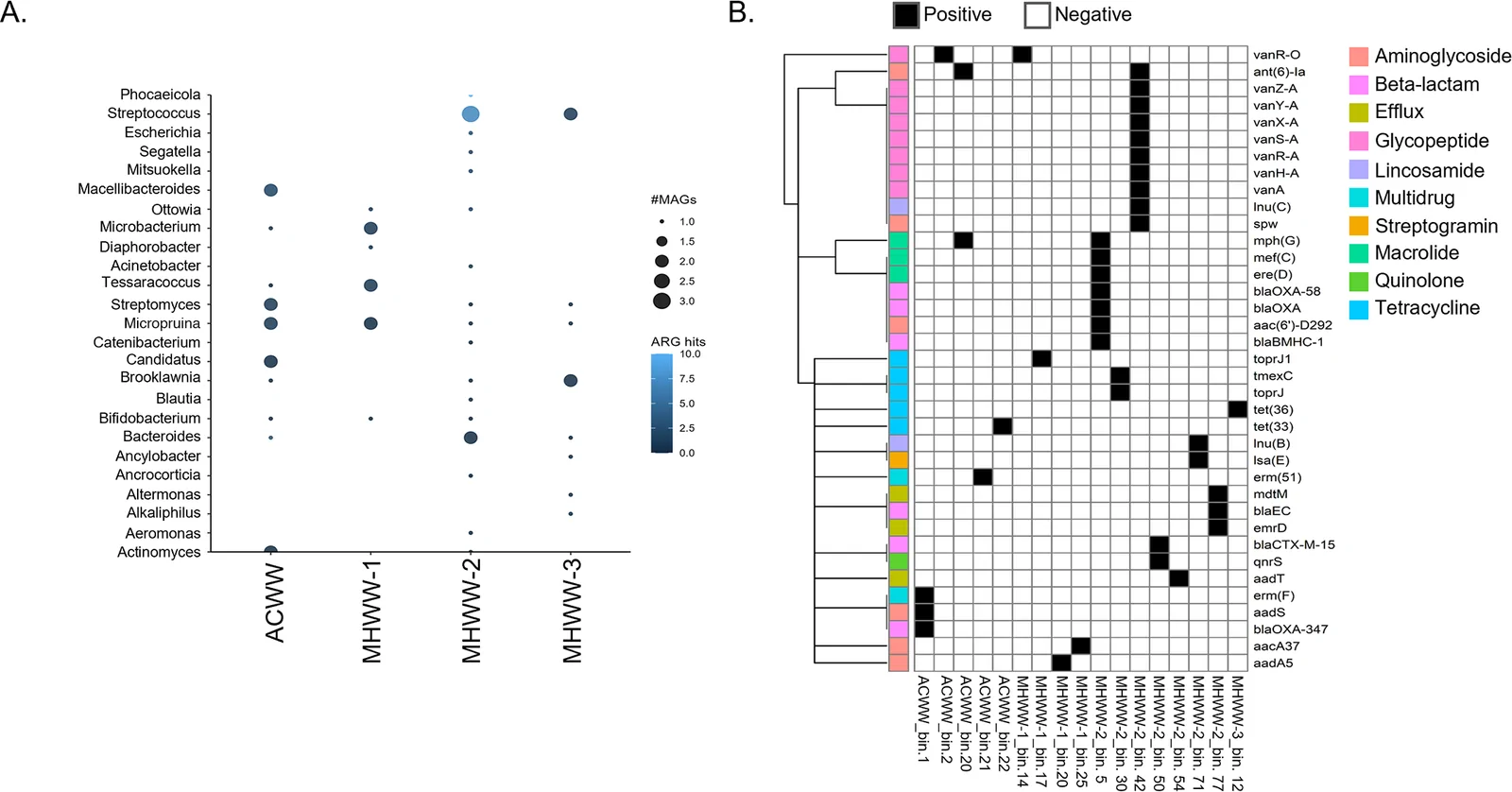
Genome-resolved distribution of antibiotic resistance genes (ARGs) across MAGs and sites. (**A**) Bubble plot summarizing ARG signal within curated MAGs. Bubble size indicates the number of MAGs recovered for each genus within a site, and color intensity indicates the total number of ARG hits detected in MAGs assigned to that genus. (**B**) Heatmap of antibiotic resistance genes across individual MAGs, clustered by ARG composition.

To identify putative hosts of ARGs, MAGs were reconstructed from the four sampling sites. Following post-binning refinement, 92 curated MAGs were retained (Table S6). Taxonomic classification encompassed 53 distinct genera, reflecting considerable genomic diversity within the wastewater microbiome (Fig. 8A). The most frequently identified genera were *Propioniciclava* and *Micropruina*, followed by *Streptococcus*, *Bacteroides*, *Streptomyces*, and *Brooklawnia*. Nonetheless, the majority of genera were rare, with 37 represented by only a single MAG. Subsequent analysis of these MAGs for ARGs identified 41 putative distinct genes across 18 MAGs, spanning 10 antibiotic classes (Fig. 8B). The highest genome-resolved resistance signal was observed in MHWW-2, which hosted seven putative ARG-positive MAGs and 30 distinct resistance genes. In contrast, ACWW and MHWW-1 harbored fewer putative ARG-positive MAGs with a more limited resistance repertoire, while MHWW-3 yielded only a single MAG associated with putative tetracycline resistance. Several putative clinically significant markers and multi-class profiles were detected within individual genomes. These included a putative *vanA*-positive MAG provisionally assigned to Bacteroidales and a MAG from MHWW-2 containing putative *blaCTX-M-15* together with *qnrS*. In addition, a *Streptococcus*-affiliated MAG from MHWW-2 contained multiple putative β-lactamase- and macrolide-associated resistance genes, while an *Escherichia*-affiliated MAG from the same site contained putative multiple efflux-associated resistance determinants (Fig. 8B). MAG-level putative ARG–VF co-carriage was identified only in a subset of bins from MHWW-2. An *Escherichia*-affiliated MAG from this site contained a putative β-lactamase together with multiple efflux-associated resistance genes and a broader putative virulence repertoire, including type 1 fimbriae, type 3 fimbriae, biofilm-associated loci, enterobactin-related iron acquisition genes, secretion-associated genes, and invasion markers. *Acinetobacte*r-affiliated MAG contained putative the efflux-associated resistance gene *aadT* together with *pilT* and *lpxC*. This finding suggested the presence of bacterial populations in HWW that combine resistance and virulence-associated traits within the same genome, potentially increasing their public health relevance. However, direct contig-level co-localization of ARGs and virulence factors was not resolved in our data set.

### Temporal dynamics of clinically important ARGs (longitudinal qPCR monitoring)

To examine the temporal pattern of clinically important ARGs in HWW, we quantified the absolute and relative abundances of 19 ARGs. Longitudinal monitoring showed temporal variation in the total ARG burden across the sampling period, alongside changes in bacterial biomass and fluctuations in several high-risk genes. In our preliminary observation, across all hospitals, the total ARG burden increased from the monsoon season to pre-winter in absolute terms, indicating a general increase in targeted ARG copy number. This trend closely mirrored the seasonal rise in bacterial biomass. Absolute ARG copies increased from 4.66 × 10² in the monsoon to 4.37 × 10⁵ in pre-winter at HWW-1, from 1.24 × 10⁴ in the monsoon to 6.22 × 10⁶ in pre-winter at HWW-2, and from 3.0 × 10¹ in the monsoon to 4.55 × 10⁵ in pre-winter at HWW-3. When normalized to bacterial biomass, the seasonal trajectory of relative abundance revealed site-specific dynamics (Fig. S2A). While HWW-1 peaked in relative burden during the post-monsoon period, HWW-2 and HWW-3 exhibited maximal relative enrichment in pre-winter. Both HWW-1 and HWW-2 demonstrated consistently high breadth, with 15 to 19 genes found over the seasons. Surprisingly, HWW-3 exhibited the most significant seasonal expansion, increasing from a restricted monsoon profile of 6/19 gene detections to broad detection in the post-monsoon and pre-winter periods, with 18/19 gene detections (Fig. S2B).

**Fig 9:**
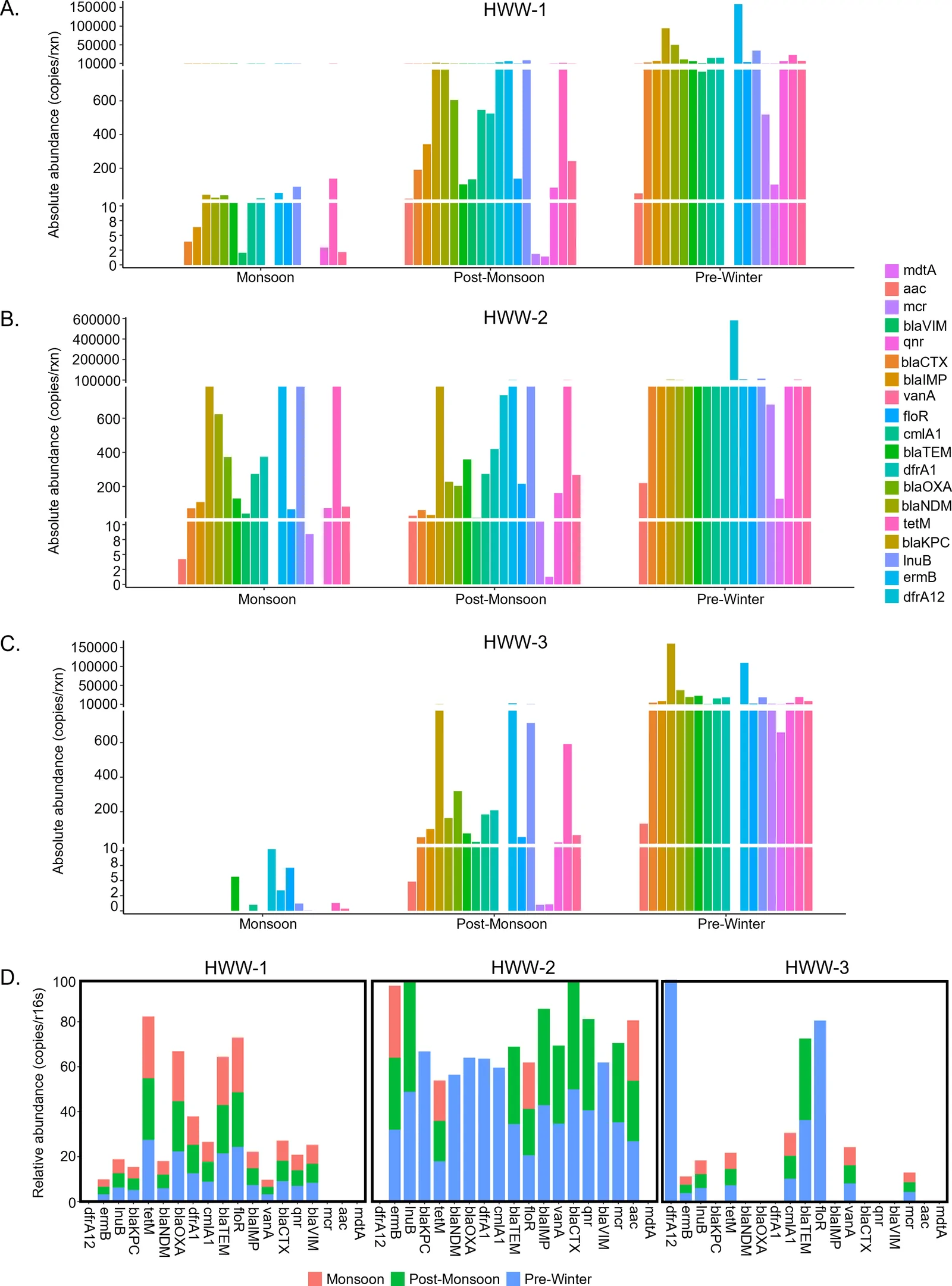
Temporal dynamics and persistence of ARGs across three hospital wastewater sites. Seasonal bar plots showing absolute abundance (copies per reaction) of individual ARGs in HWW-1 (**A**), HWW-2 (**B**), and HWW-3 (**C**) across monsoon, post-monsoon, and pre-winter. (**D**) Relative abundance (copies/16S) of the same ARG panel across seasons for each hospital site, highlighting shifts in ARG composition and enrichment.

Among the qPCR-detected ARG markers, five genes, including *blaTEM*, *ermB*, *floR*, *lnuB*, and *tetM*, were ubiquitously detected across all sites and sampling time points. This core signature suggested persistent detection of MLS, phenicol, tetracycline, and β-lactam resistance determinants independent of seasonal fluctuations. Moreover, multiple high-impact determinants also exhibited higher abundance at later sampling points. For example, carbapenemase genes such as *blaKPC* and *blaNDM* increased toward pre-winter and became dominant contributors to the absolute burden at HWW-1 and HWW-3. Several additional clinically important ARGs, such as *blaOXA*, *blaIMP*, *blaCTX*, *vanA*, *qnr*, *blaVIM*, and *mcr*, were generally present at lower baseline levels. However, they showed a preliminary pattern of increased detection frequency at 10^2^ to 10^4^ toward pre-winter in HWW-2 and HWW-3. HWW-1 showed a low-load monsoon profile that transitioned to post-monsoon enrichment dominated by MLS/tetracycline-associated genes, followed by a pre-winter absolute maximum driven by high-impact β-lactam and MLS signals. Absolute abundance increased from monsoon to post-monsoon primarily through increases in *lnuB* and *ermB*, while pre-winter was found to be dominated by *ermB*, *blaKPC,* and *blaNDM*, with elevated mid-tier contributions from *blaOXA* (Fig. 9A). Although pre-winter contained the highest absolute loads, the relative enrichment peaked in post-monsoon at this site (Fig. 9D), indicating a disproportionate rise in ARGs relative to bacterial biomass during post-monsoon. HWW-2 exhibited the highest overall ARG burden and the highest pre-winter enrichment, driven by an expansion of the trimethoprim resistance signal (Fig. 9B). While monsoon and post-monsoon displayed broad detection and high loads of the persistent core genes including *ermB*, *lnuB*, and *tetM*, pre-winter was characterized by a pronounced surge of *dfrA12*, which reached about 5.8 × 10⁶, accounting for 94% of the total ARG burden at this site. This event also corresponded to the highest relative abundance across all samples (Fig. 9D). HWW-3 showed the most apparent seasonal transition from a restricted monsoon ARG burden to a broad, high-load pre-winter profile enriched for clinically important β-lactam resistance (Fig. 9C). Monsoon samples contained only a limited subset of genes at low abundance, whereas post-monsoon introduced broad detection and increased burdens for *ermB* and carbapenemase genes. Pre-winter showed the highest measured abundance dominated by *blaKPC,* peaking at about 1.6 × 10⁵, together with high *ermB* and elevated β-lactam markers including *blaNDM* and *blaTEM*. This shift was also reflected in relative abundance, which reached its maximum in pre-winter (Fig. 9D), consistent with greater relative abundance at the pre-winter sampling point at this site.

### Temporal dynamics and characterization of MGEs (longitudinal qPCR monitoring)

To elucidate the putative mechanisms underlying the temporal expansion of the HWW resistome, we quantified the absolute and relative abundances of *intI1LC*, *intI2*, and *intI3*. The absolute abundance of the *intI1LC* varied across the sampled periods and showed a pattern similar to the total targeted ARG burden. From the monsoon to the pre-winter season, the absolute abundance of *intI1LC* increased by several orders of magnitude across all sites. In HWW-1 and HWW-2, it increased by 373-fold and 53-fold, respectively. The divergence was most pronounced in HWW-3, where the *intI1LC* copies raised from an undetectable level in monsoon to 1.5 × 10^5^ in pre-winter. The *int2* and *int3* were generally low in monsoon but became detectable and increased in post-monsoon and pre-winter (Fig. S3A and B). The *intI1LC* showed maximum relative abundance in post-monsoon season at HWW-1, but in the monsoon and pre-winter seasons at HWW-2, and increased from monsoon to post-monsoon and pre-winter at HWW-3. This pattern suggests that the relative abundance of *intI1LC* changed differently across sites and sampling periods when normalized to total bacterial signal (Fig. S3C). The proportion of class 1 integrons carrying at least one resistance gene is significantly higher in hospital samples than in other environmental sources (26). In our study, integron marker abundance showed a pattern broadly consistent with the targeted ARG burden across sites and sampling periods. In Spearman’s correlation analysis based on the longitudinal qPCR data set across three hospitals and three seasonal time points (*n* = 9), the absolute abundance of *intI1LC* was significantly correlated with the targeted ARG burden across all site-season transitions (ρ = 0.93, *P* < 0.001) (Fig. S4). This relationship may suggest that the seasonal escalation of resistance is not merely a product of community turnover, but is driven by the expansion of mobile genetic elements. Carbapenemase markers also showed strong positive associations with *intI1LC* across the longitudinal qPCR data set. Both *bla*NDM (ρ = 0.95, *P* < 0.0001) and *blaKPC* (ρ = 0.92, *P* < 0.001) were coupled with *intI1* dynamics. This may suggest preliminarily that the seasonal rise of carbapenem resistance in pre-winter is largely mediated by the horizontal dissemination or clonal expansion of integron-positive pathogens.

**Fig 10:**
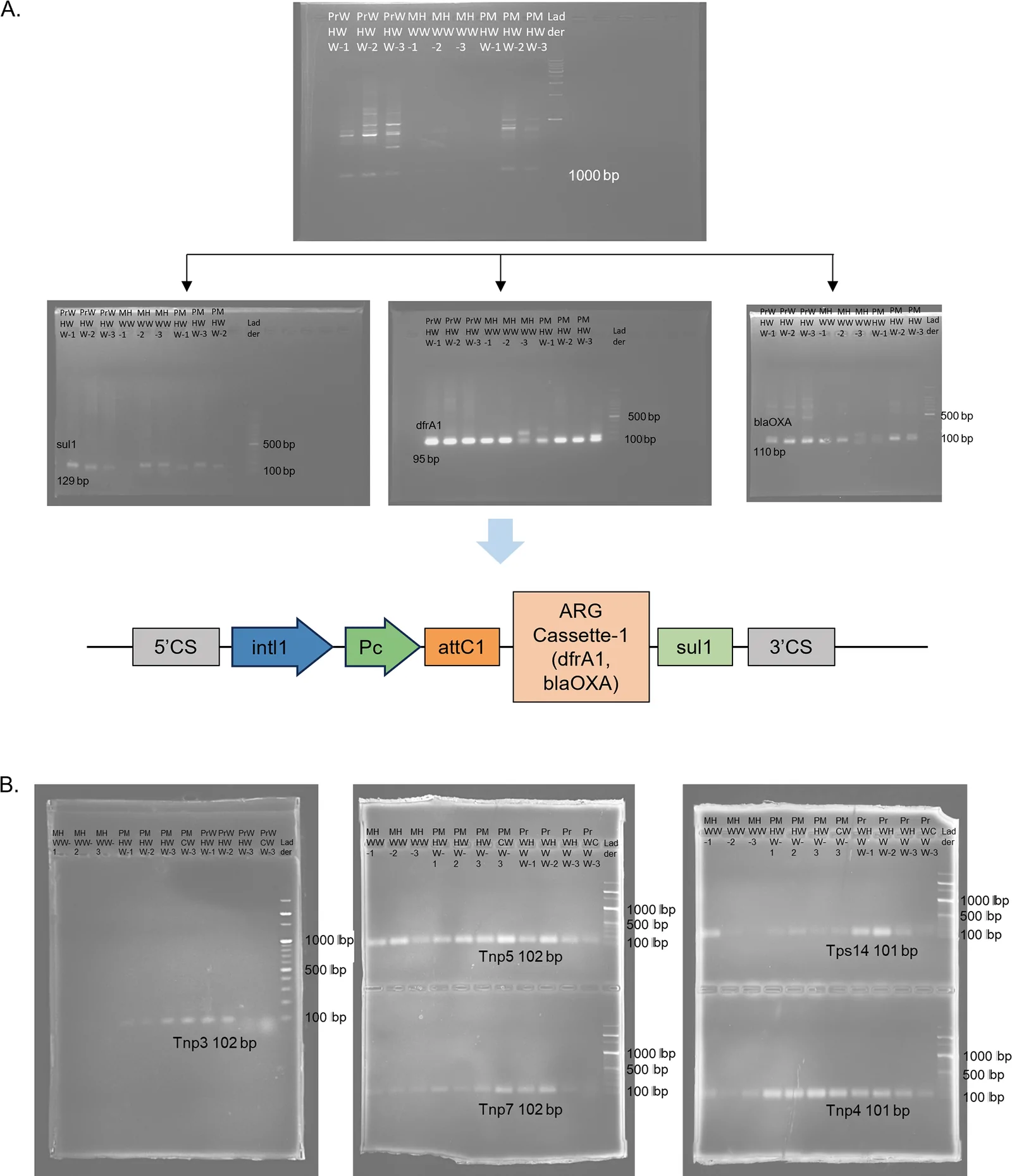
Conventional PCR screening of MGEs. (**A**) Class 1 integron-specific identification of putative gene cassette using semi-nested PCR approach and a putative model of class 1 integron in HWW. (**B**) Agarose gel electrophoresis images showing conventional PCR screening of five transposase targets (tnp3, tnp4, tnp5, tnp7, and tps14) in HWW.

Conventional PCR targeting the class 1 integron CS region was used to characterize integron carriage across seasons. All post-monsoon and pre-winter samples, as well as HWW-2 during the monsoon, yielded positive amplification with multiple bands ranging from 700 to 1,500 bp, consistent with the presence of multiple class 1 integron variants and/or heterogeneous gene cassette arrays within these communities. To further resolve cassette composition, a nested PCR strategy was applied and subsequently screened the resulting cassette amplicons for clinically important ARGs. This analysis suggested the incorporation of *sul1*, *blaOXA*, and *dfrA1* within integron gene cassette regions, supporting the role of integrons as potential carriers of clinically relevant resistance determinants in HWW (Fig. 10A). To assess additional mobility mechanisms, five transposase markers were screened. *Tnp4*, *Tnp5*, *Tnp7,* and *Tps14* were detected across all samples, indicating widespread distribution of transposon-associated mobilization potential throughout the sampling period. In contrast, Tnp3 showed a more restricted temporal distribution, being detected only in post-monsoon and pre-winter samples, suggesting seasonal enhancement of specific transposon backbones during late-season conditions (Fig. 10B). Taken together, the integron CS profiles, cassette-associated resistance markers, and transposase detections provide convergent evidence primarily that mobile genetic elements are broadly persistent but seasonally intensified, aligning with the increased ARG burdens observed during post-monsoon and pre-winter.

## DISCUSSION

In Bangladesh, HWW treatment is inadequate, and it is often discarded directly into the water bodies and mixed with community wastewater, increasing the likelihood of ARG spread to humans (10, 32, 54). In this study, we primarily demonstrated that hospital effluents carry a broader and more clinically relevant resistome than the community wastewater, including important ARGs linked to mobilizable resistance structures. Across the sampling sites, the wastewater microbiome was consistently dominated by Actinomycetota, followed by Pseudomonadota, Bacillota, and Bacteroidota. This structure is consistent with the recurring signature of HWW reported in previous studies (22, 55–57). The persistence of strict anaerobes like Faecalibacterium and Prevotella in MHWW-2 indicated a fresh effluent hotspot, where human input and limited environmental exposure may allow their detection. The methanogen *Methanothrix* in MHWW-3 signifies stagnation and sediment accumulation, resulting in anoxic microenvironments that facilitate methanogenesis, despite the drain appearing superficially aerated (58). In MHWW-1 and ACWW, the transition from fecal-derived microbes to wastewater-adapted taxa such as *Micropruina* and *Propioniciclava* may reflect ecological selection imposed by the sewer environment (59). These taxonomic patterns suggest that the HWW resistome reflects both patient-derived microbial input and local selection pressure from antibiotics and disinfectants (60, 61). The detection of gut-linked genera together with ESKAPEE pathogens in MHWW-2 supports a dominant clinical-human input signal, which is consistent with a national-scale metagenomic survey of HWW reporting enrichment of gut-associated taxa and healthcare-linked pathogens (62).

The detection of putative carbapenemase and ESBLs commonly associated with Enterobacterales indicates that these drains may serve as reservoirs for last-line antibiotic resistance. This finding is consistent with a previous culture-based study in Dhaka reporting significantly higher occurrence of metallo-β-lactamase genes in HWW (35). Our findings suggest that high-risk ARGs in wastewater are not limited to canonical pathogens, as clinically important putative ARGs in MHWW-2 were also associated with gut commensals. The frequent co-occurrence of multiple ARGs on the same contigs may indicate a co-selection mechanism, which is exposure to any one selecting agent can maintain the entire linked cassette (63). This mechanism becomes more concerning when these multi-ARG structures are carried on MGEs (64, 65). In our data, roughly one-third of putative ARGs were predicted to be plasmid-associated, and the presence of putative MGEs suggests that specific plasmid scaffolds persist as stable platforms that repeatedly capture and retain ARG modules over time (66). *VanA* is classically associated with Enterococci and is commonly carried on Tn1546-like mobile elements (67). In our study, putative *vanA*-containing contigs were consistently associated with *Enterococcus* species, whereas the MAG analysis identified a putative *vanA* cluster in a *Bacteroidales* spp.-associated bin. One possible explanation is that the *vanA* locus truly occurs in a broader host background because *Bacteroidales* are abundant gut-associated bacteria and contain ARGs (68). This would suggest that this clinically important glycopeptide resistance gene may circulate beyond its canonical reservoir. A second possibility is that the signal reflects a mobile *vanA*, where a region has been transferred from an *Enterococcus*-associated source. A third possibility is that the discrepancy may arise from a technical artifact (69). However, the finding suggested the presence of a high-risk, potentially mobilizable *vanA* resistance module in HWW. HWW was enriched for putative aminoglycoside and sulfonamide resistance and consistently showed putatively high *sul1* with *qacEΔ1*, indicating selection on integron-linked platforms compatible with disinfectant and antibiotic co-selection (64). A recent metagenomics-based study from Bangladesh reported the presence of *mcr-5* in HWW (21), which aligns with our detection of putative *mcr-5* in MHWW-2 and MHWW-3.

The ARG burden appears to correlate with hospital capacity and patient volume. MHWW-2, which possesses the highest patient load, exhibited the most diverse and abundant putative resistome, whereas MHWW-1, characterized by the lowest patient burden, showed a significantly reduced putative ARG load. This finding is consistent with several reports showing that hospital size and the intensity of clinical activity influence the ARG burden in effluent (70–72). The antibiotic consumption patterns also influence the HWW resistome. While direct prescription data were not available for this study, the enrichment of putative genes conferring resistance to aminoglycosides, macrolides, streptogramins, cephalosporins, and carbapenems may indicate high selection pressure driven by frequent clinical use of these drug classes. In a previous study, the prevalence of specific ARGs in urban sewage has been shown to correlate strongly with the volume of corresponding antibiotics prescribed within the connected healthcare facilities (73). Another study showed that phenotype-matched ARG abundance in wastewater can reflect department-level antimicrobial use (74). In this study, we found that each hospital has a putative distinct resistance profile, indicating that HWW is not a homogeneous exposure source. This inter-hospital stratification is epidemiologically significant as it indicates that an individual hospital cannot encapsulate the entire AMR risk profile of an urban healthcare network. Instead, WBE is most informative when interpreted as a comparative tool that can identify facility-specific hotspots and resistance phenotypes. The co-occurrence of elevated putative VF functional capacity with increased putative ARG load in hospital effluents supports the view that traits promoting colonization, persistence, and interbacterial interactions may contribute to the stability and dissemination potential of AMR within wastewater networks (75, 76).

In our longitudinal qPCR profiling, we show that the targeted ARGs exhibit a seasonal escalation at pre-winter across all three hospitals. Pre-winter (October–November) marks a transition toward cooler, drier conditions and is often accompanied by increased incidence of cold-associated illnesses (77). A higher clinical caseload during this period could plausibly increase antimicrobial use and patient-derived shedding, contributing to the observed late-season rise in ARG abundance (78). This finding is supported by a recent study reporting higher ARG burdens in HWW during the winter period (December–January) (37). Beyond clinical factors, reduced rainfall and weaker dilution during the drier season can concentrate microbial biomass and associated resistance determinants, producing significant increases in absolute gene copies even without proportional changes in per-cell carriage (79). In our study, the persistence of *blaTEM*, *ermB*, *floR*, *lnuB,* and *tetM* suggests sustained selection for resistance to commonly used drug classes, largely independent of season. The dramatic increase in ARG detection in HWW-3 suggests that the monsoon might mask low- and mid-abundance genes via dilution and turnover. In contrast, post-monsoon and pre-winter conditions favor persistence and detectability of rarer clinically important genes (79). In comparison, high-impact genes like *blaNDM* and *blaKPC* showed episodic behavior, peaking in the pre-winter period, underscoring the need for longitudinal surveillance to capture these transient yet high-risk peaks. WBE studies frequently detect these carbapenemase genes in hospital or urban wastewater matrices, suggesting that their pre-winter rise may reflect increased shedding of high-risk *Enterobacterales* and enhanced mobility of resistance platforms (38). Similar temporal variability in clinically important genes has been reported in short-term HWW surveillance in Finland (80).

Integrons tracked the total targeted ARG burden closely across the longitudinal qPCR data set, consistent with the established role of clinical class 1 integrons as proxies for anthropogenic pollution and as scaffolds linked to multi-resistance under human selection (81). Our data suggest an association between integron abundance and seasonal escalation of targeted ARGs. The absolute abundance of *intI1LC* showed a significant correlation with targeted ARG burden. The detection of *sul1*, *blaOXA,* and *dfrA1* within cassette amplicons further supports the view of integrons as active platforms that contribute to persistence and dissemination. Moreover, metagenomic analysis further demonstrated that clinically relevant putative ARGs, such as *blaOXA*, *blaKPC-2*, and *blaNDM-1*, were frequently co-localized with transposition and integration modules. Consistent with our findings, previous studies report that *intI1* and *sul1* abundances vary seasonally, and that 75% of elevated measurements occur in the dry season (82). Together, these findings support an association between integron abundance and the mobility potential of clinically important ARGs. However, these correlations and co-localization patterns do not by themselves demonstrate horizontal transfer events or distinguish horizontal dissemination from clonal expansion.

**Fig 11:**
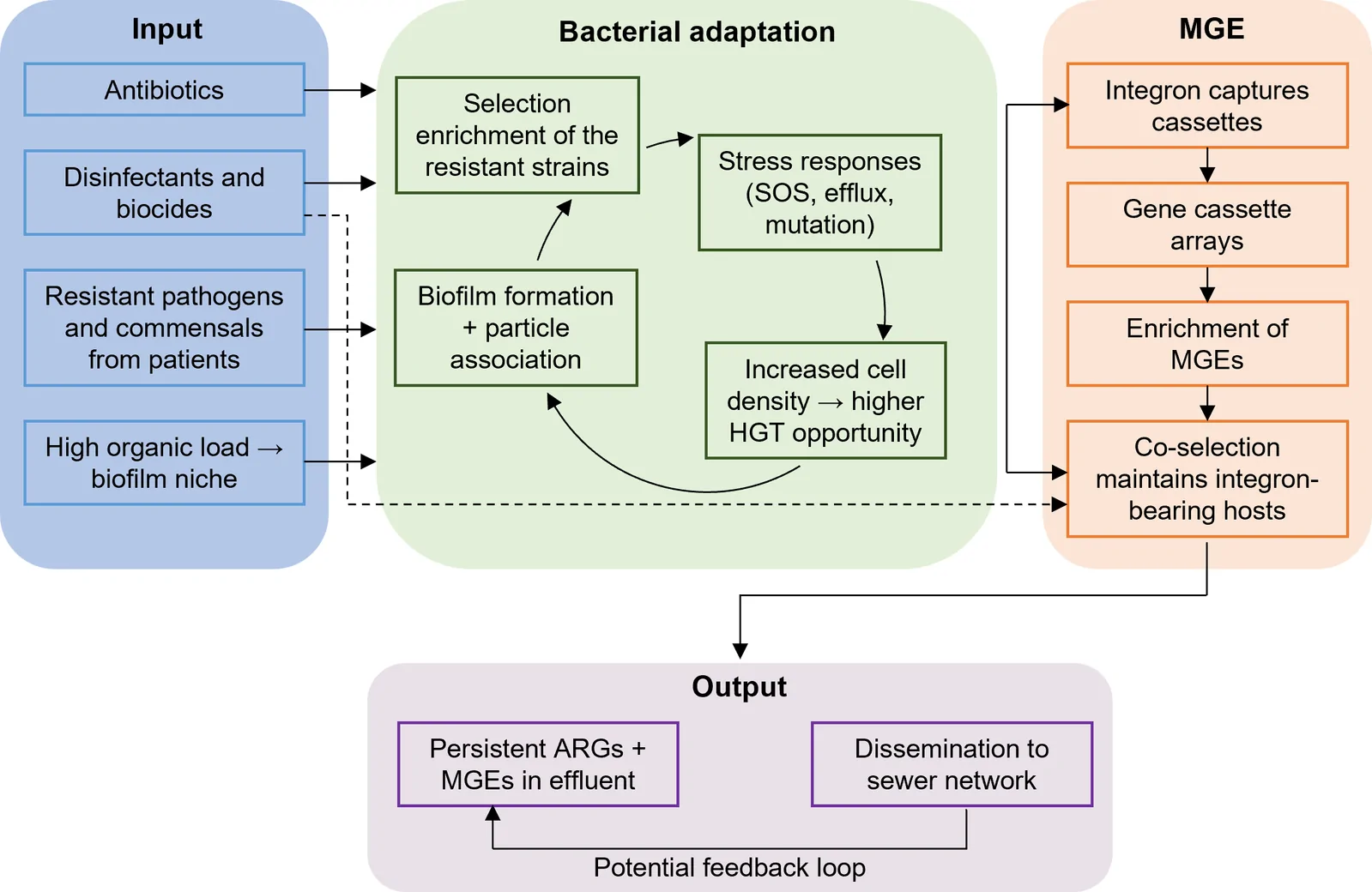
Model of ARG persistence in hospital wastewater. Continuous exposure to antibiotics and other selective agents enriches resistant bacteria and induces stress-adaptation phenotypes, creating conditions that promote horizontal gene transfer.

Our study has several limitations. First, metagenomic sequencing was conducted only at the monsoon baseline, with subsequent seasonal analysis relying on targeted qPCR. Thus, full seasonal shifts in the resistome and host were not captured. Second, short-read assembly restricted the resolution of mobile genetic elements, rendering plasmid assignments putative. In the qPCR assay, normalization to 16S rRNA genes did not account for taxon-specific copy number variations, and a representative calibration strategy was used in lieu of target-specific standard curves. Although sampling was conducted under standardized field conditions across all sites, the possible contribution of seasonal dilution and environmental mixing was not evaluated directly. Moreover, the inclusion of only one community reference makes the comparison between hospital and community wastewater remain limited in scope. To address this, further longitudinal surveillance with long-read sequencing is needed to validate these preliminary findings. Despite these limitations, our results provide several decisive extensions: (i) a comprehensive putative resistome view using metagenomics with variant-level resolution; (ii) hospital-specific effects on ARG burden; (iii) predicted plasmid and integron structures underlying clinically relevant co-resistance; and (iv) temporal patterns of clinically important ARGs (Fig. 11). Based on these, we propose a two-tier surveillance for LMIC. This framework consists of high-frequency sentinel monitoring via targeted qPCR of a core set of markers, including the anthropogenic and mobilome pair and high-consequence ARGs. This is complemented by periodic metagenomic sequencing to refresh the qPCR panel and resolve the genomic context of emerging variants. Our data further suggest that prioritizing on-site mitigation at high-signal sentinel nodes, such as HWW-2, offers a disproportionate benefit for downstream environmental protection in regions where advanced wastewater treatment is not yet ubiquitous. WBE is widely implemented in developed countries to track pathogen outbreaks and resistome signatures, but it remains limited in most developing countries. To our knowledge, this study is the first to longitudinally characterize seasonal variation of targeted ARGs in HWW in Bangladesh. By bridging the gap between high-resolution genomic data and pragmatic monitoring, this study provides a practical framework for implementing sustainable AMR surveillance.

## MATERIALS AND METHODS

### Sample collection and DNA extraction

Wastewater samples were collected from three large tertiary care hospitals, each located in a distinct region of Dhaka city. A community wastewater sample was collected from a nearby residential sewage line not directly influenced by hospital effluent (Table 1). Samples were collected over 5 months, in June, September, and November 2025, spanning three seasons, including monsoon, post-monsoon, and pre-winter. All samples were collected in the early morning (7:00–8:30 AM). At each hospital, multiple grab samples were collected from three different points within the open sewage system on the hospital premises, at an approximate depth of 0.5 m. These sub-samples were immediately combined and homogenized in a sterile 1 L bottle to create a single 1 L composite sample representative of that site. Samples were immediately placed on ice in a cooler box and transported to the laboratory, arriving within 2 h of collection. In the laboratory, each 1 L composite sample was vortexed to ensure homogeneity. A 50 mL aliquot of the unfiltered wastewater was then transferred to a sterile 50 mL centrifuge tube. To concentrate the microbial biomass, the aliquot was centrifuged at 7,000 × *g* for 30 min. Following centrifugation, the supernatant was carefully discarded. The resulting pellet, containing suspended solids and bacterial cells, was stored at −20°C until DNA extraction (22).

DNA was extracted from the stored pellets using the PureLink Microbiome DNA Purification Kit (Thermo Fisher Scientific), following the manufacturer’s protocol. The purified DNA in the microcentrifuge tube was assessed for purity and quantified. The concentration and purity of the extracted DNA were quantified using a NanoDrop 2000 Spectrophotometer (Thermo Fisher Scientific). The A_260_/A_280_ ratio was used to assess purity and detect potential protein and RNA contamination. The DNA concentration was further quantified using the Qubit dsDNA HS Assay Kit on a Qubit 4.0 Fluorometer. To evaluate DNA integrity, agarose gel electrophoresis was performed. Approximately 50 ng of DNA from each sample was loaded onto a 1% (wt/vol) agarose gel, alongside a 1 kb DNA ladder. Electrophoresis was conducted at 90 V for 1 h in 1× tris-acetic-acid-EDTA (TAE) buffer. Following electrophoresis, the gel was stained by submersion in an ethidium bromide (EtBr) solution for 30 min, then destained in distilled water. The resulting DNA bands were visualized and imaged using an AlphaImager HP System (ProteinSimple).

### Library preparation and sequencing

One hundred nanograms of genomic DNA was used as input for library preparation using the Illumina DNA Prep (M) Tagmentation Kit (Illumina, USA), following the manufacturer’s guidelines. The entire workflow was automated on the epMotion 5075 liquid handling system (Eppendorf, Germany). The final libraries were quantified using the Qubit dsDNA HS Assay Kit on a Qubit 4.0 Fluorometer. Library quality and fragment size distribution were assessed using D1000 ScreenTape on the Agilent 4150 TapeStation system. The library qualification criteria included an average size of 320 bp, a Qubit concentration >4 ng/µL, and the absence of adapter-dimer peaks below 150 bp. The quality-passed libraries were normalized to 2 nM, pooled equimolarly, and denatured following standard Illumina protocols. Shotgun metagenomic sequencing was performed on the Illumina NextSeq 2000 platform using a P2 Flow Cell with a 2 × 150 bp paired-end configuration. Raw base call (BCL) files were demultiplexed using BCL Convert to generate FASTQ files.

### Gene detection and standard preparation

Primers targeting genes including aminoglycosides (*aac*), β-lactams (*blaCTXM1*, *blaIMP*, *blaKPC2*, *blaNDM1*, *blaOXA1*, *blaTEM*, *blaVIM*), macrolide (*ermB*, *mphA-02*), phenicol (*cmlA1*, *floR*), trimethoprim (*dfrA1*, *dfrA12*), quinolone (*qnrB*), colistin (*mcr*), sulfonamide (*sul1*), tetracycline (*tetM*), and glycopeptide (*vanA*), as well as others (*lnuB-02*, *mdtA*), integrons (*intl1LC*, *intl2*, *intl3*, *intCS*), transposons (*tnpA1*, *tnpA2*, *tnpA3*, *tnpA4*, *Tp614*), and 16S rRNA (37, 83), were used in this study (Table S1). These genes were detected by conventional PCR using a thermocycler (Thermo Fisher Scientific). All PCR assays were performed in a total volume of 10 µL. Each reaction contained 0.05 µL of Go-Taq DNA Polymerase (Promega), 2 µL of 5× buffer, 0.2 µL of dNTP mixture, 1 µL of each forward and reverse primer, 2 µL of DNA template (diluted 1:20), and 3.75 µL of nuclease-free water. The PCR program was as follows: initial denaturation at 95°C for 5 min; followed by 30 cycles of 95°C for 30 s, an annealing temperature of 60°C for 1 min, and 72°C for 30 s; and a final extension at 72°C for 7 min. PCR products were visualized by electrophoresis on a 1.5% agarose gel.

Genes that tested positive in any of the nine HWW samples in the conventional PCR screening and produced a visible band of the correct amplicon size were quantified using quantitative real-time PCR (qPCR) on a QuantStudio 3 (Thermo Fisher Scientific). Standard calibration curves were generated for a representative target gene using 1:5-fold serial dilutions of purified, quantified amplicons. The concentration of stock solutions was quantified using a Nanodrop spectrofluorimeter (Thermo Fisher Scientific) (37, 84). qPCRs were performed in duplicate for each sample in a 10 µL reaction volume, and a negative control containing no template DNA was included in every run to detect and exclude any possible contamination. Each reaction contained 5 µL of KiCQStart SYBR Green qPCR ReadyMix 2× (KCQS01, Sigma-Aldrich), 1 µL of each primer, 2 µL of DNA template (5 ng), and 3 µL of nuclease-free water to a final volume. The qPCR program was as follows: 95°C for 3 min, followed by 40 cycles of denaturation at 95°C for 15 s, annealing at 60°C for 60 s, and elongation at 72°C for 20 s. Melt curve analysis was performed after each qPCR run at 95°C for 5 min and 65°C for 1 min to confirm the specificity of the amplification. The melting peak specificity was confirmed using the QuantStudio Design and Analysis Software 2 (Thermo Fisher Scientific). The size of all qPCR amplicons was further confirmed by 1.5% agarose gel electrophoresis. Reaction efficiencies and regression coefficients were determined for all standard curves. As standard curves were generated for a single representative target gene rather than for each individual assay, absolute quantification for the remaining targets should be interpreted as approximate rather than fully target-calibrated. Samples showing near-identical melting curves with a sharp peak and ct < 31 were considered positive. Here, 31 was identified as the detection limit. The mean ct value was used to calculate gene copies, and the relative copy number was calculated by dividing the ct value for the gene of interest by that of the 16S rRNA gene using the following equations (85).


$$\;\;\;\;\;\;\;\;\;\text{ Absolute abundace of genes }=10^{\frac{31-ct}{\frac{10}{3}}}$$

$$\;\;\;\;\;\;\;\;Reative\;abundance=\frac{Absolute\;abundance}{16s\;Gene\;Copies}$$

### Integron characterization

To characterize integrons, the class 1 integron variable region was amplified using the CS primer set by conventional PCR, as previously described. Cycling conditions were as follows: initial denaturation at 95°C for 5 min; 35 cycles of denaturation at 95°C for 1 min, annealing at 55°C for 1 min, and extension at 72°C for 5 min, with the extension step increased by 5 s per cycle; and a final extension at 72°C for 10 min (83). Amplicons were verified by agarose gel electrophoresis and purified using the Wizard SV Gel and PCR Clean-Up System (Promega) according to the manufacturer’s instructions. Purified DNA was stored at −20°C until further use. The purified integron amplicons were subsequently used as templates for PCR screening of cassette-associated resistance genes, including *sul1*, *aac*, *blaOXA*, and *dfrA1*, using the same reaction mixture and cycling conditions as described earlier.

### Bioinformatics analysis

Quality assessment of the raw reads was performed using FastQC v0.11.9 and MultiQC v1.9 to evaluate base-call quality distribution, the percentage of bases above Q20 and Q30, GC content, and adapter contamination (https://www.bioinformatics.babraham.ac.uk/projects/fastqc/). Low-quality bases (Phred score < 20), ambiguous bases (N), and adapter sequences were trimmed using fastp v0.20.1 (86), retaining reads ≥ 50 bp. Approximately 54, 61, 53, and 46 million reads were obtained for MHWW-1, MHWW-2, MHWW-3, and ACWW, respectively. High-quality reads were assembled using MEGAHIT v1.2.9 with parameters *--k-min 35 --k-max 141 --k-step 28* (87). Contigs shorter than 200 bp were excluded from subsequent analysis. Assembly quality was evaluated using Bowtie2 v2.5.4 and Samtools v1.22.1 by mapping reads back to contigs, and assembly statistics (N50, GC%, total contigs) were summarized using QUAST v5.2.0 (22, 88). Taxonomic classification was performed using Kraken2 v2.1.3 and Bracken against the Kraken standard database (October 2025 version), containing RefSeq complete bacterial genomes/proteins (89, 90). Kraken2 reports were filtered to remove taxa that accounted for <0.1% of the total classified reads.

To determine the abundance of ARGs directly from the cleaned reads, ShortBRED v0.9.5 was employed (91). A set of specific ARG markers was generated using the *shortbred_identify.py* module, utilizing antibiotic resistance protein sequences from the Comprehensive Antibiotic Resistance Database (CARD) (https://card.mcmaster.ca) as the input, with the parameters: *-clustid* 0.95 *-ref* Uniref90 (22). The relative abundance of AMR gene families was quantified by mapping quality-filtered reads to the generated markers using *shortbred_quantify.py*. To ensure robust detection and reduce noise, stringent post-quantification filtering was applied; hits were excluded if they had fewer than two mapped reads or a mean reads per kilobase per million (RPKM) less than 0.001.

Assembled contigs were taxonomically classified using Kraken2 and assigned lineages using TaxonKit v0.2.4 (22). Contigs classified as Eukaryota were removed. Filtered prokaryotic contigs were annotated with Prokka v1.14.6 using metagenome setting (92). ARGs were identified using RGI v6.0.5 (https://github.com/arpcard/rgi) against CARD v3.2.7 with local settings. ARG abundance was estimated by mapping paired-end reads to the assembled contigs using Bowtie2 v2.5.4 and processing alignments with Samtools v1.22.1; abundances were normalized as RPKM. Plasmid- versus chromosome-derived AMR contigs were predicted using PlasFlow v1.1 and PlasClass (score ≥ 0.7 for plasmids), and plasmid contigs were further screened with Abricate v1.0.1 (https://github.com/tseemann/abricate) against PlasmidFinder (93). MGEs were identified using mobileOG-db (94), and virulence factors were detected with Abricate v1.0.1 against the Virulence Factor Database (VFDB).

To reconstruct microbial genomes from metagenomic data, raw paired-end reads were assembled *de novo* using MEGAHIT v1.2.9 with *--k-min 35*, *--k-max 141*, *--k-step 28*, and minimum contig length of 1,000 bp (87). Reads were mapped to contigs with Bowtie2 v2.5.4, and alignments were converted, sorted, and indexed using Samtools v1.22.1 (95). Contig depths were computed with *jgi_summarize_bam_contig_depths* and used for binning with MetaBAT2 v1.7 using *-minContig-Length 1500* (96). Bin quality was assessed with CheckM v1.2.4, retaining bins with ≥50% completeness and <10% contamination (22). MAGs were taxonomically assigned using Mash v2.3 against NCBI RefSeq by selecting the closest reference genome (97). AMR and VF determinants in high-quality MAGs were identified using AMRFinder v4.0.23 with the *--plus* option and Abricate v1.0.1 against VFDB (98).

### Statistical analysis

Co-occurrence patterns between ARGs and bacterial taxa were explored using the top 20 ARG subtypes and genus-level taxonomic abundances across the four baseline metagenomic samples. ARG abundance was obtained from the RGI-derived RPKM matrix, and taxonomic abundance was derived from Kraken outputs summarized at the genus level. Spearman’s rank correlation was used to identify positive ARG–genus associations with concordant abundance trends. The resulting exploratory network was visualized in Cytoscape v3.9.1. All analyses were conducted in R v4.2 using pavian, ggplot2, ggpubr, pheatmap, cowplot, vegan, phyloseq, and ggvegan.

## Data Availability

All the data have been provided in the manuscript or in the supplementary file. Additional data can be given upon request. The sequences are available in the National Center for Biotechnology Information (NCBI) database under the BioProject PRJNA1399457 and SRA accession numbers SRR36711353, SRR36711354, SRR36711355, and SRR36711356.

## SUPPLEMENTAL MATERIAL

Supplemental table and figures spectrum.00755-26-s0001.docx Table S1 and Fig. S1 to S4.

Table S2 spectrum.00755-26-s0002.xlsx Resistance genes identified using ShortBRED method.

Table S3 spectrum.00755-26-s0003.xlsx Resistance genes identified using RGI.

Table S4 spectrum.00755-26-s0004.xlsx Plasmid identified using PlasmidFinder.

Table S5 spectrum.00755-26-s0005.xlsx Identified virulence factors using Abricate against the Virulence Factor Database.

Table S6 spectrum.00755-26-s0006.xlsx MAG Assignment to ARG and VFDB.

ASM does not own the copyrights to Supplemental Material that may be linked to, or accessed through, an article. The authors have granted ASM a non-exclusive, world-wide license to publish the Supplemental Material files. Please contact the corresponding author directly for reuse.
