# Supplementary material for: Metagenomic profiling and temporal dynamics of antimicrobial resistance genes across hospital wastewaters in Dhaka, Bangladesh
Source: Microbiol Spectr. 2026 Oct 1;14(11):e00755-26. doi: 10.1128/spectrum.00755-26 (PMC13729169; doi:10.1128/spectrum.00755-26)
Supplement: Supplementary file 1 [file spectrum.00755-26-s0001.docx]

**Table S1:** Primer used in this study

| **Gene name** | **Strand** | **Sequence** | **Amplicon size** |
| --- | --- | --- | --- |
| *16s rRNA* | F | GGGTTGCGCTCGTTGC | Variable |
|  | R | ATGGYTGTCGTCAGCTCGTG |  |
| *aac* | F | CCCTGCGTTGTGGCTATGT | 56 |
|  | R | TTGGCCACGCCAATCC |  |
| *blaCTXM1* | F | GGAGGCGTGACGGCTTTT | 63 |
|  | R | TTCAGTGCGATCCAGACGAA |  |
| *blaIMP* | F | AAGTTAGTCAATTGGTTTGTGGAGC | 269 |
|  | R | CAAACCACTACGTTATCTGGAGTGTG |  |
| *blaKPC2* | F | CAGCTCATTCAAGGGCTTTC | 196 |
|  | R | GGCGGCGTTATCACTGTATT |  |
| *blaNDM1* | F | ATTAGCCGCTGCATTGAT | 154 |
|  | R | CATGTCGAGATAGGAAGTG |  |
| *blaOXA1* | F | CGGATGGTTTGAAGGGTTTATTAT | 110 |
|  | R | TCTTGGCTTTTATGCTTGATGTTAA |  |
| *blaTEM* | F | AGCATCTTACGGATGGCATGA | 101 |
|  | R | TCCTCCGATCGTTGTCAGAAGT |  |
| *blaVIM* | F | GCACTTCTCGCGGAGATTG | 135 |
|  | R | CGACGGTGATGCGTACGTT |  |
| *cmlA1* | F | TAGGAAGCATCGGAACGTTGAT | 101 |
|  | R | CAGACCGAGCACGACTGTTG |  |
| *ermB* | F | TAAAGGGCATTTAACGACGAAACT | 172 |
|  | R | TTTATACCTCTGTTTGTTAGGGAATTGAA |  |
| *floR* | F | ATTGTCTTCACGGTGTCCGTTA | 61 |
|  | R | CCGCGATGTCGTCGAACT |  |
| *mcr* | F | ATGACAAAAATCTTGATGATTGAAGAT | 175 |
|  | R | CCATCATAGGCAATCCTAAATCCA |  |
| *dfrA1* | F | GGAATGGCCCTGATATTCCA | 95 |
|  | R | AGTCTTGCGTCCAACCAACAG |  |
| *qnrB* | F | GGMATHGAAATTCGCCACTG | Variable |
|  | R | TTYGCBGYYCGCCAGTCGAA |  |
| *sul1* | F | CAGCGCTATGCGCTCAAG | 129 |
|  | R | ATCCCGCTGCGCTGAGT |  |
| *tetM* | F | CATCATAGACACGCCAGGACATAT | 101 |
|  | R | CGCCATCTTTTGCAGAAATCA |  |
| *vanA* | F | AAAAGGCTCTGAAAACGCAGTTAT | 150 |
|  | R | CGGCCGTTATCTTGTAAAAACAT |  |
| *dfrA12* | F | CCTCTACCGAACCGTCACACA | 85 |
|  | R | GCGACAGCGTTGAAACAACTAC |  |
| *mdtA* | F | CCTAACGGGCGTGACTTCA | 70 |
|  | R | TTCACCTGTTTCAAGGGTCAAA |  |
| *mphA-02* | F | TGATGACCCTGCCATCGA | 63 |
|  | R | TTCGCGAGCCCCTCTTC |  |
| *lnuB-02* | F | AAAGGAGAAGGTGACCAATACTCTGA | 104 |
|  | R | GGAGCTACGTC AAACAACCAGTT |  |
| *int1* | F | CGAACGAGTGGCGGAGGGTG | 312 |
|  | R | TACCCGAGAGCTTGGCACCCA |  |
| *int2* | F | TGCTTTTCCCACCCTTACC | 195 |
|  | R | GACGGCTACCCTCTGTTATCTC |  |
| *int3* | F | GCCACCACTTGTTTGAGGA | 138 |
|  | R | GGATGTCTGTGCCTGCTTG |  |
| *int1LC* | F | GCCTTGATGTTACCCGAGAG | 196 |
|  | R | GATCGGTCGAATGCGTGT |  |
| *tnpA1* | F | CATCATCGGACGGACAGAATT | 113 |
|  | R | GTCGGAGATGTGGGTGTAGAAAGT |  |
| *tnpA2* | F | GGGCGGGTCGATTGAAA | 108 |
|  | R | GTGGGCGGGATCTGCTT |  |
| *tnpA3* | F | AATTGATGCGGACGGCTTAA | 102 |
|  | R | TCACCAAACTGTTTATGGAGTCGTT |  |
| *tnpA4* | F | CCGATCACGGAAAGCTCAAG | 101 |
|  | R | GGCTCGCATGACTTCGAATC |  |
| *Tp614* | F | GGAAATCAACGGCATCCAGTT | 101 |
|  | R | CATCCATGCGCTTTTGTCTCT |  |
| *CS-1* | F | GGCATCCAAGCAGCAAG | Variable |
|  | R | AAGCAGACTTGACCTGA |  |

**Figure S1:** Taxonomic reservoirs of ARG-associated ORFs across community and hospital wastewater sites. A) Phylum-level distribution of ARG; B) Genus-level heatmap of the number of unique ARGs detected per genus across sites; color intensity indicates higher ARG richness within a genus.

**Figure S2:** Seasonal patterns of ARG abundance across hospital wastewater sites. A) Heatmap of 16S-normalized relative abundance (gene copies/16S copies) for 19 ARG targets across three hospital wastewater sites. B) Heatmap of absolute abundance (copies per reaction) for the same genes and samples. MHWW, PMHWW and PrWHWW labels denote monsoon, post-monsoon and pre-winter collections, respectively.

**Figure S3:** Temporal persistence and seasonal dynamics of integrons across three hospital wastewater sites. A) Absolute abundance. B) Seasonal trajectories of integron in absolute abundance. C) Relative abundance.

**Figure S4:** Spearman Correlation depict the association of temporal dynamics among the antibiotic resistance genes and integrons.
